# Application of a recombinant novel trypsin from *Trichinella spiralis* for serodiagnosis of trichinellosis

**DOI:** 10.1186/s13071-023-06067-7

**Published:** 2024-01-04

**Authors:** Lu Lu Han, Qi Qi Lu, Yang Li Li, Wen Wen Zheng, Pian Ren, Ruo Dan Liu, Jing Cui, Zhong Quan Wang

**Affiliations:** Department of Parasitology, Medical College, Zhengzhou University, Zhengzhou, 450001 China

**Keywords:** Trichinellosis, *Trichinella spiralis* trypsin, Recombinant antigen, Serodiagnosis, ELISA

## Abstract

**Background:**

The excretory/secretory (ES) antigen of *Trichinella spiralis* muscle larvae (ML) is currently the most widely used diagnostic antigen to detect *T. spiralis* infection. However, this antigen has certain drawbacks, such as a complicated ES antigen preparation process and lower sensitivity during the early phase of infection. The aim of this study was to investigate the features of a novel *T. spiralis* trypsin (TsTryp) and evaluate its potential diagnostic value for trichinellosis.

**Methods:**

The TsTryp gene was cloned and recombinant TsTryp (rTsTryp) expressed. Western blotting and an enzyme-linked immunosorbent assay (ELISA) were performed to confirm the antigenicity of rTsTryp. The expression pattern and distribution signature of TsTryp at various life-cycle stages of *T. spiralis* were analyzed by quantitative PCR, western blotting and the immunofluorescence test. An ELISA with rTsTryp and ML ES antigens was used to detect immunoglobulins G and M (IgG, IgM) in serum samples of infected mice, swine and humans. The seropositive results were further confirmed by western blot with rTsTryp and ML ES antigens.

**Results:**

TsTryp expression was observed in diverse *T. spiralis* life-cycle phases*,* with particularly high expression in the early developmental phase (intestinal infectious larvae and adults), with distribution observed mainly at the nematode outer cuticle and stichosome. rTsTryp was identified by *T. spiralis*-infected mouse sera and anti-rTsTryp sera. Natural TsTryp protease was detected in somatic soluble and ES antigens of the nematode. In mice infected with 200 *T. spiralis* ML, serum-specific IgG was first detected by rTsTryp-ELISA at 8 days post-infection (dpi), reaching 100% positivity at 12 dpi, and first detected by ES-ELISA at 10 dpi, reaching 100% positivity at 14 dpi. Specific IgG was detected by rTsTryp 2 days earlier than by ES antigens. When specific IgG was determined in serum samples from trichinellosis patients, the sensitivity of rTsTryp-ELISA and ES antigens-ELISA was 98.1% (51/52 samples) and 94.2% (49/52 samples), respectively (*P* = 0.308), but the specificity of rTsTryp was significantly higher than that of ES antigens (98.7% vs. 95.4%; *P* = 0.030). Additionally, rTsTryp conferred a lower cross-reaction, with only three serum samples in total testing positive from 11 clonorchiasis, 20 cysticercosis and 24 echinococcosis patients (1 sample from each patient group).

**Conclusions:**

TsTryp was shown to be an early and highly expressed antigen at intestinal *T. spiralis* stages, indicating that rTsTryp represents a valuable diagnostic antigen for the serodiagnosis of early *Trichinella* infection.

**Graphical Abstract:**

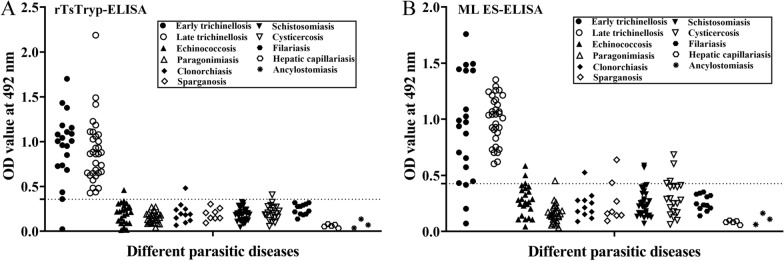

**Supplementary Information:**

The online version contains supplementary material available at 10.1186/s13071-023-06067-7.

## Background

Trichinellosis is an important zoonotic parasitosis caused by nematodes of the genus *Trichinella*. In humans, *Trichinella* infection occurs through the ingestion of raw or semi-cooked meat of animals infected with *Trichinella* larvae, with *Trichinella spiralis* the prime causative agent [1, 2]. This parasitic disease can occur worldwide, with 50 trichinellosis outbreaks reported in the European Union between 2015 and 2019 [3] and 13 trichinellosis outbreaks involving 1604 cases and 12 deaths were recorded in Southeast Asian countries from 2001 to 2021 [4]. In China, specifically, eight trichinellosis outbreaks were documented during the 2009–2020 period, with 479 patients and two deaths reported [5]. The consumption of undercooked pork and pork products are the predominant source of *T. spiralis* infection. Trichinellosis therefore remains a thorny challenge and threat to public health and food safety [6].

Following the ingestion of *Trichinella*-infected meat, muscle larvae (ML) are liberated and develop into intestinal infectious larvae (IIL) in the small intestine, where they undergo molting 4 times to ultimately develop into adult worms (AW). Following copulation, the mature females produce newborn larvae (NBL), which penetrate the skeletal muscles through the circulatory and lymphatic systems and develop into the encapsulated ML [7]. The clinical manifestations of trichinellosis at these different development stages differ. The initial intestinal stage of the infection is often accompanied by nonspecific gastrointestinal symptoms, including nausea, emesis, abdominal pain and diarrhea. The major symptoms and signs at the muscular stage (acute stage) are fever, periorbital edema, myalgia or muscle weakness, skin eruption, among others [8, 9]. Clinical diagnosis of trichinellosis is rather difficult due to its non-pathognomonic symptomatology in the initial stage of infection, especially in sporadic cases and upon presentation of early cases of a new outbreak. Moreover, medical practitioners from non-endemic areas are usually unfamiliar with the manifestations of trichinellosis, and patients are often not taken seriously and/or misdiagnosed.

The definitive diagnosis of trichinellosis can be established on the basis of epidemiological investigation (focusing on a history of raw or undercooked meat consumption), muscle biopsy and serological testing. However, muscle biopsy is not suitable for patients with a mild infection and/or during early infection [10]. At the present time, *T. spiralis* ML excretory/secretory (ES) antigen-based serological assays are extensively applied for the detection of the infection, as recommended by the International Commission on Trichinellosis (ICT) and the Food and Agriculture Organization of the UN (FAO)/WHO/World Organization for Animal Health (OIE) [11, 12]. Nevertheless, the main drawbacks of this ML ES antigen are a low sensitivity during the early infection period due to a 3- to 5-week-long window between *Trichinella* infection and seroconversion for positivity. Seroconversion in patients with trichinellosis often occurs between the third and fifth weeks post-infection [13, 14]. In addition to exposure level (e.g. the infectious dose), stage-specific antigens used in the immunodiagnosis are also an important factor affecting the detection of *Trichinella-*specific immunoglobulin G (IgG). Recently, the presence of circulating antigen and DNA in serum samples as alternative markers for detecting *T. spiralis* infection have received increasing attention. However, some studies showed that the levels of free antigens circulating in the blood are generally low due to the existence of immune complexes. The authors of one study reported that the overall positive rate of circulating antigen in patients with clinical trichinellosis was only 30–50% and, in addition, the persistent period of* T. spiralis* DNA was transitory in infected host’s blood [15]. Therefore, it is necessary to explore novel detection/diagnostic antigens to achieve a simple, accurate, reliable and early diagnosis of trichinellosis. An enzyme-linked immunosorbent assay (ELISA) with ML ES antigens has demonstrated superior performance in terms of sensitivity as well as an acceptable specificity and reproducibility; it has been widely applied to the serological diagnosis of trichinellosis [12, 16]. However, the ES antigens prepared from the ML were found to be stage specific and may not contain epitopes identified by the antibodies produced at the intestinal stage [17]. At the enteral phase of *T. spiralis* infection, surface and ES proteins from IIL and AW are expressed early during infection to simulate the host’s immune system and trigger production of enteral *Trichinella*-specific antibodies. Therefore, the surface and ES proteins from the IIL and AW might contain the early intestinal stage-specific expressed protein molecule markers for early diagnosis of trichinellosis [18, 19].

 In one study, *T. spiralis* adult crude antigens were probed by serum from infected swine or mice 7 days post-infection (dpi) [20]. Recombinant proteins from IIL or pre-adults have been identified by serum from infected swine as early as 15–20 dpi [21, 22]. *Trichinella spiralis*-specific IgG in infected mouse sera was detected by indirect ELISA with AW and IIL ES antigens at 8–10 dpi, but not detected by ELISA with the ML ES antigens prior to 12 dpi. Moreover, the ELISA with these worm ES antigens also had low false positivity with infection serum of other parasites [23, 24]. Therefore, novel diagnostic antigens for trichinellosis need to be further characterized and developed, with a focus on worms of the intestinal stage (IIL and AW) of *T. spiralis* [25, 26].

Additionally, the preparation of the ML ES antigens is cumbersome and time-consuming, requiring first the collection of ML from *T. spiralis*-infected laboratory animals and then their culture to prepare ES antigens in vitro [27, 28]. Although ES antigens are less complex than worm somatic crude antigens, they still have the potential to cross-react with other parasitic helminths [29]. Thus, the ICT recommended that positive results of ELISA with ML ES antigens need to be further confirmed by western blot [12]. Recently, the preparation of recombinant antigens using DNA recombinant technology has proved to be time-saving and relatively cost-effective and to have a good reproducibility. Therefore, recombinant *Trichinella* antigens have the potential to be serodiagnostic antigens for trichinellosis [30–32].

In preliminary studies, we identified a novel highly expressed trypsin of *T. spiralis* (TsTryp, GenBank: XM_003381619.1) in IIL ES antigens using sera from infected mice at 8–10 dpi and sera from patients with early trichinellosis [26, 33, 34]. TsTryp is a likely to be a potential early diagnostic antigen. In the present study we aimed to further determine the biological properties of TsTryp and evaluate its prospective serodiagnostic values for early trichinellosis.

## Methods

### Worm and animals

The *T. spiralis* isolate used in this study (ISS534) was obtained from an infected domestic pig in Henan province of China [35]. In our laboratory, this nematode was passaged in mice at 6-month intervals. For the present study, we bought 20 female BALB/c mice (age: 8 weeks; weight: 20–25 g each) from Henan Provincial Medical Laboratory Animal Center (Zhengzhou, China). All of the mice were raised in individual ventilated cage under the specific-pathogen-free (SPF) condition, and each mouse was orally infected with 200 *T. spiralis* ML.

### Serum samples

Blood samples (100 μl) were collected from the tail vein of each of the 20 mice infected with 200 *T. spiralis* ML on alternate days between 0 and 30 dpi [31]. Serum samples were collected from mice infected with 200 ML of *T. spiralis* (T1), *Trichinella nativa* (T2), *Trichinella britovi* (T3), *Trichinella pseudospiralis* (T4) and *Trichinella nelsoni* (T7) at 35 dpi. Sera from mice infected with *Angiostrongylus cantonensis*, *Capillaria hepatica*, *Schistosoma japonicum* and* Spirometra mansoni* plerocercoids and from *Toxoplasma gondii* were collected in our laboratory or were a gift from our Chinese colleagues. Fifty individual serum samples from uninfected healthy mice were used as the negative serum control. Eighteen large white pigs were inoculated orally with 5000 ML; infected pigs were sacrificed at 70 dpi, and infected serum (swine infection serum) was obtained. Serum samples from swine naturally infected with *Ascaris suum* were a present from Prof. Ming Xin Song (Northeast Agricultural University, China).

Fifty-two serum samples were obtained from 52 patients treated for trichinellosis during two trichinellosis outbreaks that occurred in Yunnan province, southwestern China in 2003 (20 patients [age range: 12–65 years; 12 males]; serum collected at 19 dpi) and 2013 (32 patients [age range: 15–70 years; 25 males]; serum collected at 35 dpi). All patients had a history of consuming raw or undercooked meat and showed typical clinical trichinellosis manifestations, including fever, myalgia, periorbital or facial edema and eosinophilia. All patients tested positive for *Trichinella*-specific IgG antibodies by the ML ES antigen ELISA; muscle biopsy was performed in two trichinellosis patients at 35 dpi and encapsulated ML were found [8, 24]. All serum samples of these trichinellosis patients were tested again in our laboratory by ELISA with ML or AW ES antigens; the anti-*Trichinella* IgG was positive [24]. The etiological *Trichinella* species of two trichinellosis outbreaks was not identified. Since only one species *T. spiralis* of the *Trichinella* genus was found in southwestern China, the causative agent of the two trichinellosis outbreaks is likely to be *T. spiralis* [36]. Serum samples from patients with other parasite infections were conserved in our laboratory as follows: five cases with hepatic capillariasis, three cases with ancylostomiasis (*Ancylostoma duodenale*), 12 cases with lymphatic filarialsis (*Wuchereria bancrofti*), 25 cases with paragonimiasis (*Paragonium skrjabini*), 11 cases with clonorchiasis (*Clonorchis sinensis*), 30 cases with schistosomiasis (*Schistosoma japonicum*), eight cases with sparganosis (*Spirometra mansoni* sparganum), 20 cases with cysticercosis (*Taenia solium* cysticercus) and 24 cases with cystic echinococcosis (*Echinococcus granulosus*). The confirmatory diagnosis of the patients with parasitic diseases was made on the basis of serological tests and fecal parasitological examination [24, 37]. For comparison, 100 serum samples form healthy individuals residing in non-trichinellosis endemic areas were collected and tested as a negative result for the parasite infection tests mentioned above.

### Collection of worms and its antigens

Murine muscles infected with *T. spiralis* at 42 dpi were artificially digested and the ML were obtained as previously reported [38]. The IIL and AW were isolated from the intestines of infected mice at 6 h post-infection (hpi) and 3 and 6 dpi, respectively [39, 40]. Female AW at 6 dpi were first washed in phosphate-buffered saline (PBS), then cultivated at 37 °C for 18 h in RPMI 1640 medium (Sangon, Shanghai, China) supplemented with 100 μg/ml streptomycin and 200 U/ml penicillin, following which NBL were recovered [27, 41]. Somatic soluble proteins (crude antigens) from various *T. spiralis* developmental phases, including ML, IIL, AW and NBL, were prepared as described previously [42, 43]. The ML ES antigens were prepared according to a previously reported method [23]. Briefly, the ML were washed throughly in sterile normal saline solution and serum-free RPMI-1640 medium containing 200 U penicillin/ml and 100 μg streptomycin/ml, and then cultivated at 5000 ML/ml in the same culture medium under 5% CO_2_ at 37 °C for 18 h. After cultivation, the medium containing the ES proteins were filtered through a 0.2-μm membrane into a 50-ml conical tube and then centrifuged at 5000 *g*, 4 °C for 2 h. The supernatant was filtered once again through an Amicon Ultra 3K centrifugal filter. The protein concentration of ML ES antigens determined by the Bradford assay was 1.29 mg/ml. The filtered supernatant was preserved at − 80 °C until use.

### Analysis of TsTryp sequence and phylogeny

The NCBI GenBank was searched for the peptide sequence of TsTryp (GenBank: XM_003381619.1). The physicochemical characteristics, functional domain, transmembrane domain and signal peptide of TsTryp were analyzed and predicted by the bioinformatics online websites ExPasy, NCBI, SignalP 5.0 and TMHMM [44]. The tertiary structure and enzyme active sites of TsTryp were predicted using CN3D and PyMOL software [45]. Phylogenetic analysis of amino acid sequences of TsTryp and of trypsin of other *Trichinella* species was performed using MEGA7.0 software, and their phylogeny was constructed on the basis of neighbor-joining (NJ) method [46, 47]. The GenBank accession numbers of trypsin’s peptide sequence from different *Trichinella* species and other organisms are as follows: *T. nativa* (KRZ48719.1), *T. britovi* (KRY59262.1), *T. psendospiralis* (KRY77859.1), *T. murrelli* (KRX44632.1), *Trichinella* T6 (KRX79013.1), *T. nelsoni* (KRX20911.1), *Trichinella* T8 (KRZ96745.1), *Trichinella* T9 (KRX62817.1), *T. papuae* (KRZ66153.1), *T. patagoniensis* (KRY20428.1), *T. zimbabwensis* (KRZ13763.1), *Trichuris suis* (KFD57430.1), *Trichuris trichiura* (CDW57755.1), *Caenorhabditis elegans* (NP_501379.2), *Necator americanus* (XP_013293798.1), *Ancylostoma caninum* (RCN34081.1), *Anisakis simplex* (VDK43792.1), *Brugia malayi* (XP_042934028.1), *Steinernema carpocapsae* (TKR57687.1), *Homo sapiens* (XP_011526282.1) and *Mus musculus* (P69525.1).

### Preparation of recombinant TsTryp and anti-recombinant TsTryp serum

The RNAs of IIL were isolated using TRIzol (Sangon). The TsTryp coding sequence of 1956 bp was amplified by PCR with specific primers: (i) 5′-CCGAATTCCTACCAGATGACAA ATGTGGATT-3′ containing the restriction enzymatic digestion site of *EcoR*I (underlined); and (ii) 5′-TCGTCGACTCAAAGTGTCTG AAAACGGGAAC-3′ containing the restriction enzymatic digestion site of *Sal*I (underlined). The pET-32a plasmid was used to link PCR products and to construct the recombinant expression plasmid pET-32a/TsTryp, which were transferred into *Escherichia coli* BL21 (DE3). Subsequently, the pET-32a/TsTryp plasmid was induced for 6 h at 25 °C with 0.8 mM IPTG as inducer [48]. Recombinant TsTryp (rTsTryp) and thioredoxin (TRX) protein were purified using the Ni–NTA Sefinose Resin Kit (BBI Life Sciences Corp., Shanghai, China) [45]. rTsTryp and its antigenicity were analyzed by western blot as reported before [49].

Thirty female mice were immunized by subcutaneous injection with rTsTryp (20 mice, 20 μg/mouse) and TRX tag protein (10 mice, 20 μg/mouse) emulsified with complete Freund’s adjuvant. Boost immunization with rTsTryp and TRX protein emulsified in incomplete Freund’s adjuvant at a 1:1 ratio was administered twice with a 2-week interval between boosters [44, 50]. At 2 weeks after the last immunization, blood samples were obtained via tail-vein bleeding, and anti-rTsTryp and anti-TRX immune sera were isolated; the antibody level of anti-rTsTryp and anti-TRX IgG was detected by conventional indirect ELISA using rTsTryp and TRX protein (2.0 μg/ml) [51]. The specific IgG titer of both anti-rTsTryp and anti-TRX IgG was 1:10^5^, and the optical density (OD) value of anti-rTsTryp and anti-TRX IgG was 1.33 ± 0.53 (*n* = 20) and 1.20 ± 0.32 (*n* = 10), respectively. Serum samples were also collected from uninfected normal mice prior to rTsTryp immunization; these samples were considered to be the pre-immune serum.

### Western blot

rTsTryp, ML crude antigens and ES antigens were analyzed using sodium dodecyl sulfate-polyacrylamide gel electrophoresis (SDS-PAGE), and the protein products were electrophoretically transferred to a nitrocellulose (NC) membrane (MerckMillipore, Burlington, MA, USA). For blocking, the membranes were placed in non-fat milk (5%) dissolved in Tris-buffer saline containing 0.05% Tween (TBST) at 37 °C for 2 h. The strip was then probed with the different sera mentioned above (infected serum, anti-rTsTryp serum, anti-TRX serum and pre-immune serum) at 1:200 overnight at 4 °C [42]. After rinsing in TBST, the strip was incubated with horse radish peroxidase (HRP)-conjugated anti-mouse/swine/human IgG as the secondary antibodies, respectively (1:10,000 dilutions; SouthernBiotech., Birmingham, AL, USA) at room temperature for 1 h. Finally, staining was performed with the substrate 3, 3′-diaminobenzidine (DAB; Sigma-Aldrich, St Louis, MO, USA) [52, 53]. To determine the expression levels of TsTryp at diverse *T. spiralis* stages, the strips were visualized by enhanced chemiluminescence (ECL) using the ultrasensitive ECL chemiluminescence reagent kit (ECL; Meilunbio, China) and the bands then analyzed using ImageJ software [54].

### Quantitative PCR

*Trichinella spiralis* at different developmental stages were collected and their total RNAs were extracted using TRIzol reagent (Sangon). The PrimeScript RT Reagent Kit (TaKaRa, Kyoto, Japan) was used to reverse transcribe the RNAs into complementary DNA (cDNA) [55, 56]. The SYBR Green PCR Master Mix (Takara) was used for quantitative PCR (qPCR), using the TsTryp primer sequences for qPCR (5′-GGAGCTGAATCATCTTATGG-3′ and 5′-CGTCTATCTGCTTTACCACC-3′). The expression level of TsTryp messenger RNA (mRNA) was normalized to that of *T. spiralis* glyceraldehyde 3-phosphate dehydrogenase (GAPDH; GenBank: AF452239) as the internal reference. Each assay was carried out in triplicate. Relative quantification of TsTryp was analyzed by the 2^−ΔΔCt^ method [57].

### Immunofluorescence test

Natural TsTryp expression on the surface of cuticles during the different *T. spiralis* worm phases and tissue localization were investigated by the immunofluorescence test (IFT) on entire worms as well as worm cross-sections [58, 59]. Briefly, fresh worms at diverse stages were fixed in 4% paraformaldehyde followed by embedding. Serial 3-µm-thick worm sections were cut and fixed on glass slides. Sections and whole worms were sealed at room temperature for 2 h using PBS supplemented with 5% normal goat serum, followed by washing in PBS before being probed for 10 h at 4 °C with infection serum, anti-rTsTryp serum, pre-immune serum and anti-TRX serum, respectively, diluted at 1:20. On the second day, the treated parasites and cross-sections were washed in PBS, then stained by FITC-goat anti-mouse IgG conjugate (Santa Cruz Biotechnology, Dallas, TX, USA) at 1:100 dilutions, at 37 °C in dark for 1 h, and examined by fluorescent microscopy (Olympus, Tokyo, Japan) [60, 61].

### Assay of anti-*Trichinella *antibodies by ELISA

The optimal coating concentration of rTsTryp and ML ES antigens, dilution of serum samples and conjugates were first assessed by the checkerboard titration method, as reported previously [62]. The titration results revealed that 2.0 μg/ml of rTsTryp and ES antigens, respectively, was the optimal coating concentration for detecting specific IgG and immunoglobulin M (IgM); the optimal dilutions of mouse, swine and human serum were 1:100 to detect IgG and 1:50 to detect mouse IgM; the optimal conjugate dilution was 1:10,000 dilutions for HRP-conjugated anti-human/mouse/swine IgG; and 1:5000 dilutions were optimal for HRP-anti-mouse IgM conjugate. The ELISA was used to assay anti-*Trichinella* antibodies as mentioned previously [8, 37]. In brief, the microtiter plate (Corning Inc., Corning, NY, USA) was coated using rTsTryp and ML ES antigens, which were diluted in carbonate bicarbonate buffer (0.05 M, pH 9.6) and incubated overnight at 4 °C. The plate was blocked using 5% skimmed milk diluted in PBS containing 0.05% Tween 20 (PBST) and probed at 37 °C for 2 h with various serum samples (mouse, swine and human sera). After three rinses in PBST, the plate was incubated at 37 °C for 1 h with HRP-conjugated anti-human/mouse/swine IgG or anti-mouse IgM (Southern Biotech.) diluted in PBST. Following further washes, coloration was performed with the substrate* O*-phenylenediamine dihydrochloride (OPD; Sigma-Aldrich), which was dissolved in phosphate citric acid buffer containing 0.2 M Na_2_HPO_4_ and 0.1 M citric acid and supplemented with H_2_O_2_, for 30 min; the reaction was stopped with 2 M H_2_SO_4_ (50 μl/well). The OD values (absorbance at 492 nm) were measured using a microplate reader (Tecan Austria GmbH, Grödig, Austria). The cutoff value was defined as 2.1-fold the mean OD value of negative control sera of 50 uninfected mice, 55 uninfected pigs or 100 healthy persons [63]. The cutoff values for rTsTryp-ELISA mouse serum IgG and IgM were 0.241 and 0.266, respectively, and those of the ES antigens-ELISA were 0.256 and 0.270, respectively. The cutoff values of the rTsTryp-ELISA and ES antigens-ELISA were 0.271 and 0.281, respectively, for testing swine serum IgG and 0.357 and 0.426, respectively, for testing human serum IgG.

### Statistical analysis

The data were analyzed using SPSS version 22.0 software (SPSS IBM Corp., Armonk, NY, USE). Normality and homogeneity of data variances was checked prior to performing analysis of variance (ANOVA). Data were first assessed for normal distribution by the Shapiro–Wilk test. Under the test standard *α* = 0.05, *P* > 0.05 indicated that the data were subject to normal distribution. The homogeneity of data variances was also checked by homogeneity of variance test in one-way ANOVA; *P* > 0.05 was considered to be consistent with homogeneity of variance. The results are presented as the mean ± standard deviation (SD). Expression levels of rTsTryp at diverse *T. spiralis* worm stages were analyzed by one-way ANOVA. The sensitivity and specificity of rTsTryp and ML ES antigens were compared by the Chi-square test (*χ*^2^). *P-*values < 0.05 were accepted as indicating statistical significance.

## Results

### Biological features of TsTryp

**Fig. 1:**
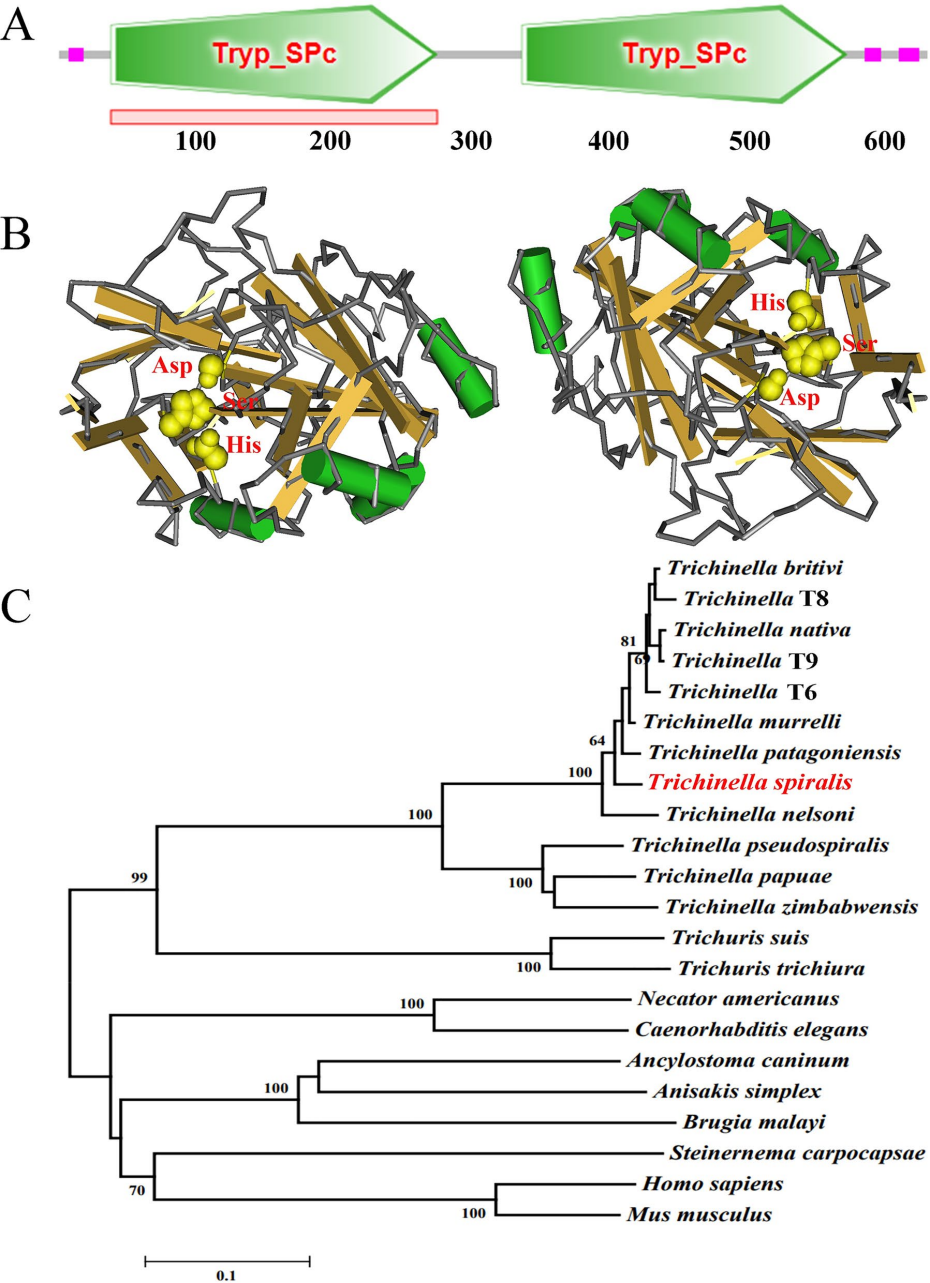
Tertiary structure of TsTryp and phylogeny of trypsin in 22 organisms by NJ method. **a** Online analysis of the NCBI and SMART databases showed that TsTryp had two identical and independent trypsin-like serine protease domains (Tryp_SPc). **b** The NCBI-Cn3D online website was used to conduct homology modeling for TsTryp and predict its tertiary structure. The secondary structure of TsTryp comprises alpha-helices (green cylinders), beta-strands (brown sheets) and random coils (gray strands). TsTryp had two similar trypsin-like domains; the three enzyme active sites of Asp, Ser and His are shown as yellow spheres. **c** Phylogenetic tree of the trypsin of *Trichinella spiralis* and other nematodes were constructed by NJ method in MEGA7.0 software. NJ, Neighbor-joining; TsTryp, *T. spiralis* trypsin

The TsTryp cDNA encoding 667 amino acid residues comprises 2004 bp. This protein has a molecular weight of 71.6 kDa and isoelectric point (pI) of 8.83. The N terminal of TsTryp has a typical hydrophobic structure, and all 67 amino acids are outside of the membrane. Alignment analysis of the amino acid sequence of TsTryp with those of the trypsin of eight encapsulated *Trichinella* species (*T. patagoniensis*,* T. nelsoni*,* T. murrelli*,* Trichinella* T6, *T. nativa*,* T. britovi*,* Trichinella* T8 and *Trichinella* T9) revealed 94.5%, 94.2%, 94.0%, 94.0%, 93.9%, 93.7%, 93.5% and 93.4% similarity, respectively. Also, a similarity of 75.6%, 75.4% and 71.4% between TsTryp and the trypsin from three non-encapsulated * Trichinella* species (*T. papuae, T. zimbabwensis* and *T. pseudospiralis*), respectively, was also found (Additional file 1: Figure S1). Prediction of the subcellular localization of TsTryp revealed that TsTryp had an extracellular localization and might be part of secretory protein. Moreover, prediction of TsTryp structure suggested that TsTryp had two independent and similar trypsin-like domains and that there were three active sites (Asp, Ser and His) in each domain (Figs. 1a, b).

**Fig. 2:**
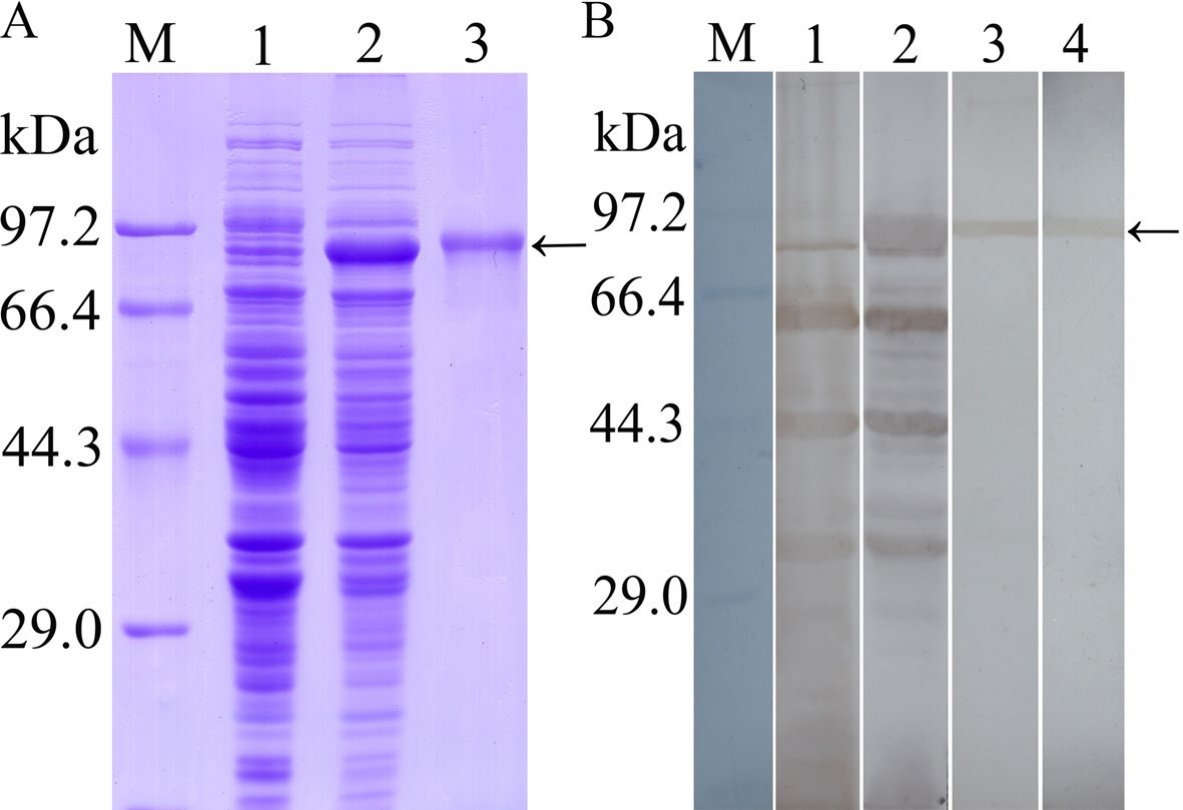
Identification of rTsTryp. **A** SDS-PAGE of rTsTryp. Lanes: M, Protein marker; 1, lysates of non-induced BL21 containing the pET-32a/TsTryp plasmid; 2, lysates of induced BL21 with pET-32a/TsTryp; 3, purified rTsTryp. **b** Western blot of rTsTryp. Lanes: M, Protein marker; 1, lysates of non-induced pET-32a/TsTryp was not identified by serum of infected mice (murine infection serum); 2, lysates of pET-32a/TsTryp following induction were identified using murine infection serum; 3, 4, purified rTsTryp probed using murine infection serum (lane 3) and monoclonal antibody to His tag (lane 4), respectively. Arrow indicates the rTsTryp protein band of 91.6 kDa. BL21, *Escherichia coli* strain BL21; rTsTryp, recombinant *T. spiralis* trypsin; SDS-PAGE, sodium dodecyl sulfate-polyacrylamide gel electrophoresis

The phylogeny of TsTryp with trypsins from other nematodes is shown in Fig. 1c. Specifically, the phylogenetic tree demonstrates that the genus *Trichinella* is monophyletic, with TsTryp having a relatively closer genetic relationship with the enteral parasitic nematodes of the genus *Trichuris* (*T. suis* and *T. trichiura*). Two clear clades can be seen in the genus *Trichinella*: an encapsulated clade (*T. patagoniensis*,* T. nelsoni*,* T. murrelli*, *Trichinella* T6, *T. nativa*, ,* T. britovi*, *Trichinella* T8, *Trichinella* T9) and a non-encapsulated clade (*T. papuae*, T*. zimbabwensis*, *T. pseudospiralis*).

### Expression and identification of rTsTryp

The TsTryp gene was cloned and the rTsTryp protein expressed. After induction with IPTG, the TRX-His + TsTryp fusion protein was expressed in *E. coli* BL21 carrying the pET-32a/TsTryp plasmid. SDS‒PAGE of the purified rTsTryp showed that rTsTryp had an apparent single protein band of 91.6 kDa, which corresponded to the predicted size, including the 71.6-kDa TsTryp and the approximately 20-kDa TRX + His-tag (Fig. 2a). The results were further validated by rTsTryp being recognized by *T. spiralis* infection serum on the western blot (Fig. 2b).

**Fig. 3:**
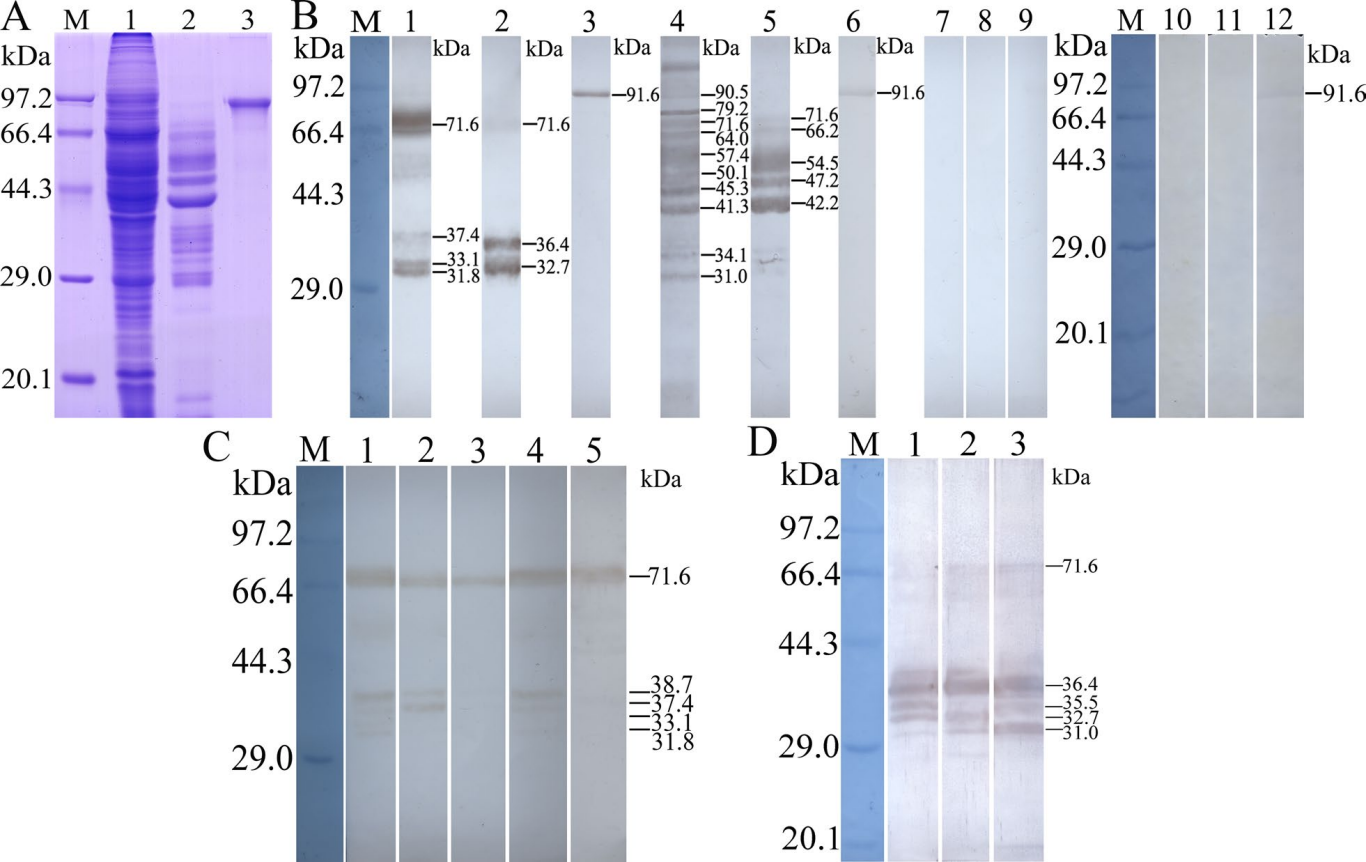
Antigenicity analysis of rTsTryp. **a** SDS-PAGE of rTsTryp. Lanes: M, Protein marker; 1, muscle larvae crude antigens; 2, muscle larvae ES antigens; 3, purified rTsTryp. **b** Western blot of rTsTryp. Lanes: M, Protein marker; 1, 2, 3, natural TsTryp in muscle larva crude antigens (lane 1), ES antigens (lane 2) and rTsTryp (lane 3), identified by anti-rTsTryp serum; 4–9, natural TsTryp in muscle larva crude antigens (lane 4) and ES antigens (lane 5) and rTsTryp (lane 6) probed using infection serum, but not probed using uninfected murine serum (lanes 7, 8, 9, respectively); 10, 11, 12, natural TsTryp in muscle larva crude antigens (lane 10) and ES antigens (lane 11) not recognized by anti-TRX serum, but rTsTryp was recognized by anti-TRX serum (lane 12). **c** Western blot of natural TsTryp in crude antigens of muscle larvae, IIL, 3 dpi adult worms, 6 dpi adult worms and NBL (lanes 1–5, respectively), identified using immune serum against rTsTryp. **d** Natural TsTryp in ES antigens of muscle larvae, IIL and 3 dpi adult worms (lanes 1, 2, 3, respectively) were identified using immune serum against rTsTryp by western blot. ES, Excretory/secretory; IIL, intestinal infective larvae; NBL, newborn larvae; rTsTryp, recombinant *T. spiralis* trypsin; SDS-PAGE, sodium dodecyl sulfate-polyacrylamide gel electrophoresis; TRX, thioredoxin

Following separation of the soluble antigens and ES antigens of ML (Fig. 3a), western blot analysis showed that native TsTryp of 31.8–71.6 kDa in the crude soluble antigens of ML were detected by anti-rTsTryp serum while the infected serum detected the native proteins of 31.0–90.5 kDa. In ES antigens of the ML, natural TsTryp of 32.7–71.6 kDa was detected by anti-rTsTryp serum, while infection serum identified natural proteins of 42.2–71.6 kDa. However, the crude and ES antigens were not identified by anti-TRX serum and uninfected normal serum (Fig. 3b). Native TsTryp of 71.6 kDa in crude antigens of ML, IIL, AW at 3 and 6 dpi (3-/6-day AW) and NBL was also observed by using anti-rTsTryp serum (Fig. 3c). Several forms of natural TsTryp of 31–71.6 kDa from ML, IIL and 3-day AW ES antigens were also identified by anti-rTsTryp serum (Fig. 3d). The results demonstrated that native TsTryp was expressed during diverse *T. spiralis* life-cycle phases and that TsTryp and its different isoforms belonged to the group of secretory proteins.

### Expression level of TsTryp mRNA and protein at various* T. spiralis* developmental stages

**Fig. 4:**
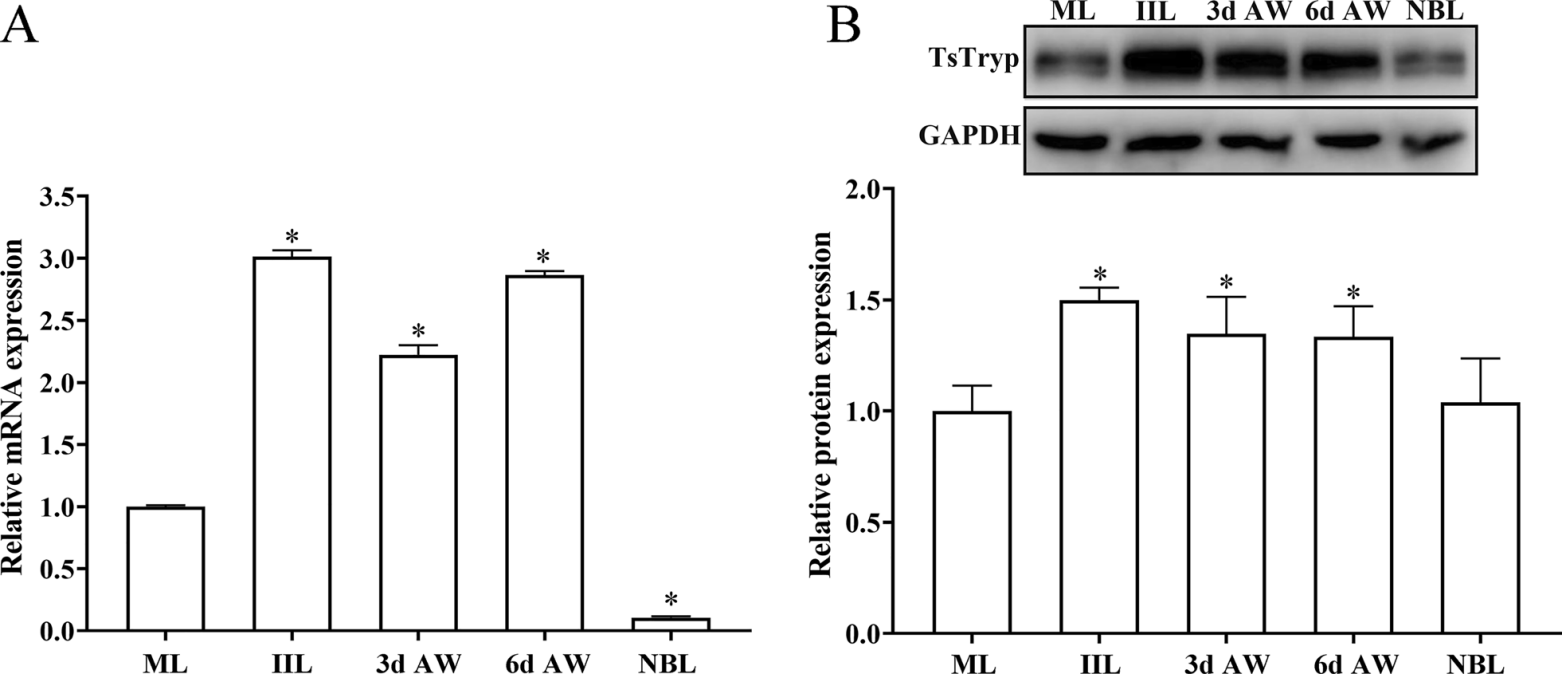
Expression of TsTryp at different developmental stages of *T. spiralis*. **a** qPCR test for TsTryp transcription level at different *T. spiralis* stages, calculated based on the 2^−ΔΔCt^ method. The fold changes in TsTryp gene level were normalized to the respective GAPDH control. **b** Western blot of TsTryp protein expression level during various worm stages. TsTryp expression in crude antigens of ML larvae, IIL, 3-/6 dpi AW and NBL was assayed by western blot using immune serum against rTsTryp. The data were derived from three separate tests. Asterisk indicates a statistical significant difference at *P < 0.05 compared to the ML stage. AW, Adult worms; d, day post-infection; ES, excretory/secretory; GAPDH, glyceraldehyde 3-phosphate dehydrogenase; h, hour post-infection; IIL, intestinal infective larvae; ML, muscle larvae; mRNA, messenger RNA; NBL, newborn larvae; qPCR, quantitative PCR; rTsTryp, recombinant *T. spiralis* trypsin

The qPCR data demonstrated that the TsTryp gene was expressed during all life-cycle stages of *T. spiralis*, including ML, IIL, 3-/6-day AW and NBL (Fig. 4a). The expression level of TsTryp mRNA clearly differed at the different phases (*F* = 2365.300, *P* < 0.0001). Notably, the mRNA level of TsTryp at the IIL stage was 3.01-fold higher than that at the ML stage (*F* = 4412.472, *P* < 0.0001), and the TsTryp transcriptional level in 3-day-/6-day AW was also remarkably higher than that of the ML (2.22- and 2.87-fold higher, respectively; *F*_3d AW_ = 724.522, *F*_6d AW_ = 9166.032, *P* < 0.0001). The western blot results showed that the expression level of TsTryp in the IIL and 3-/6-day-old AW was significantly higher than that at the ML stage (1.50- 1.35- and 1.33-fold higher, respectively; *F*_IIL_ = 45.751, *P* = 0.002;* F*_3d AW_ = 8.910, *P* = 0.041; *F*_6d AW_ = 10.415, *P* = 0.032) (Fig. 4b). These results demonstrated that TsTryp was expressed during all life-cycle stages of the nematode and that TsTryp was a highly expressed protein early during the IIL and AW stages; consequently, it is likely an early diagnostic molecular marker.

### Expression and localization of TsTryp in various* T. spiralis* life-cycle phases

**Fig. 5:**
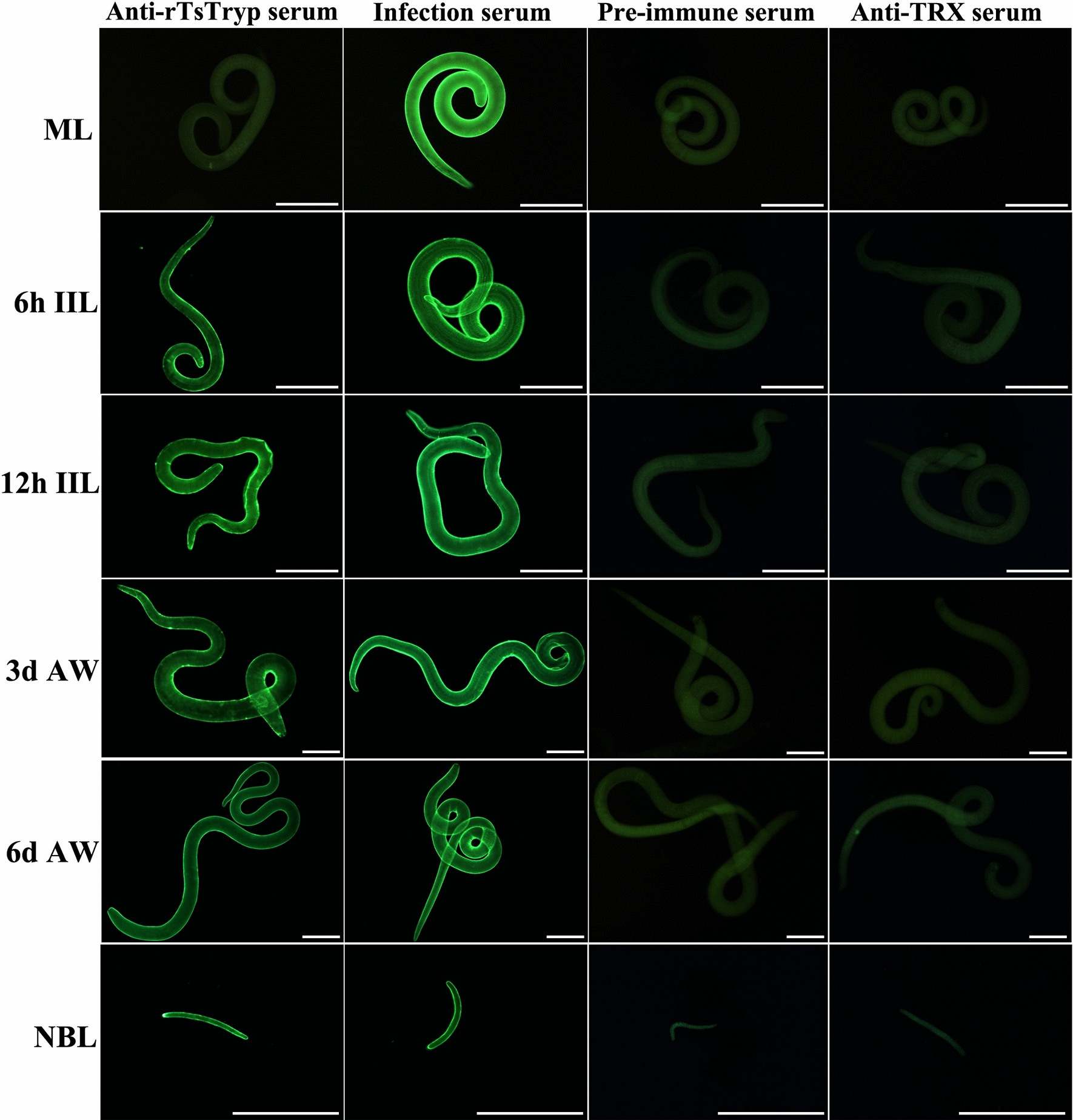
Expression of TsTryp in the epicuticle of diverse developmental stages of *T. spiralis* by IFT. Entire intact worms were incubated with immune serum against rTsTryp, and a green immunofluorescence signal was observed on the outer cuticle of *T. spiralis* at diverse life-cycle phases, including 6- and 12-hpi IIL, 3- and 6-dpi AW and NBL, but not ML. Pre-immune serum and anti-TRX serum did not detect any immunofluorescence signal in the worms. *Trichinella-*infected murine serum was applied as a positive control of reproducibility. Scale bars: 50 μm (ML, 6- and 12-hpi IIL); 100 μm (3- and 6-dpi AW); 25 μm (NBL). AW, Adult worms; IFT, immunofluorescence test; IIL, intestinal infective larvae; ML, muscle larvae; NBL, newborn larvae; rTsTryp, recombinant *T. spiralis* trypsin; TRX, thioredoxin

**Fig. 6:**
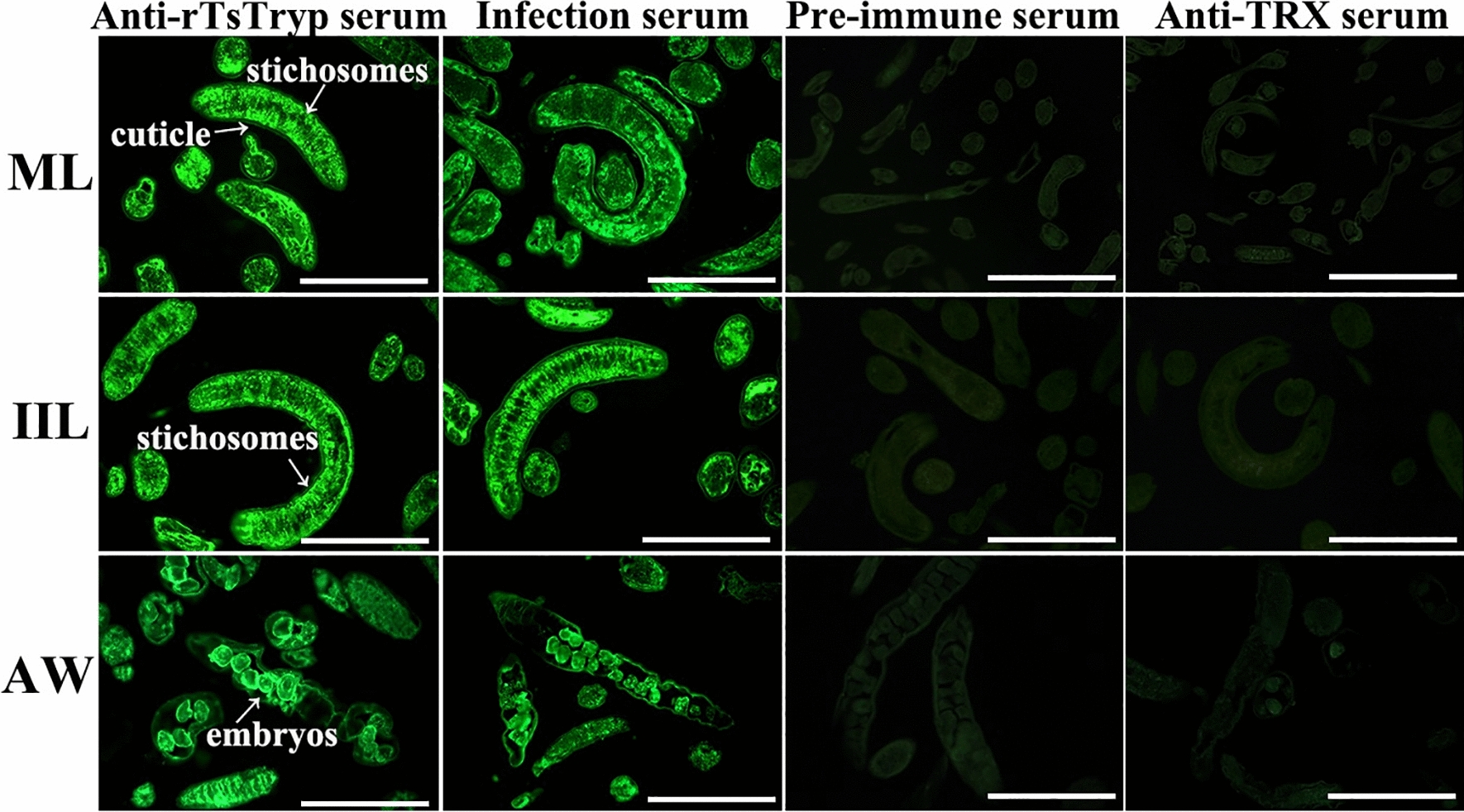
Localization of TsTryp in *T. spiralis* worm tissue by IFT*.* Green immunofluorescence signal was detected in the epidermis and stichosome and around the uterus of female embryos. No immunofluorescence was probed by pre-immune serum and anti-TRX serum in worm cross-sections. Scale bars: 50 μm. AW, Adult worm; IFT, immunofluorescence test; IIL, intestinal infective larvae; ML, muscle larvae; rTsTryp, recombinant *T. spiralis* trypsin

The expression and distribution pattern of TsTryp in the surface of the whole worm and within the worm body was determined by the IFT with anti-rTsTryp serum. A clear green immune fluorescence signal was detected in the epicuticle of * T. spiralis* at different developmental phases (6- and 12-h IIL, 3- and 6-day AW, NBL), with the exception of the ML (Fig. 5). The results of the IFT with anti-rTsTryp serum on the prepared tissue sections suggested that immune fluorescence was present in the cuticle, stichosome and uterus of female embryos (Fig. 6). No immune fluorescence staining was detected on whole worms or cross-sections using normal serum and anti-TRX serum. Taken together, these results demonstrated that TsTryp is a surface and secretory protein of this parasite and that it is mainly expressed on the surface of intestinal worms (IIL and AW) and their stichosome.

### Quality assessment of rTsTryp and ES antigens by Western blot

**Fig. 7:**
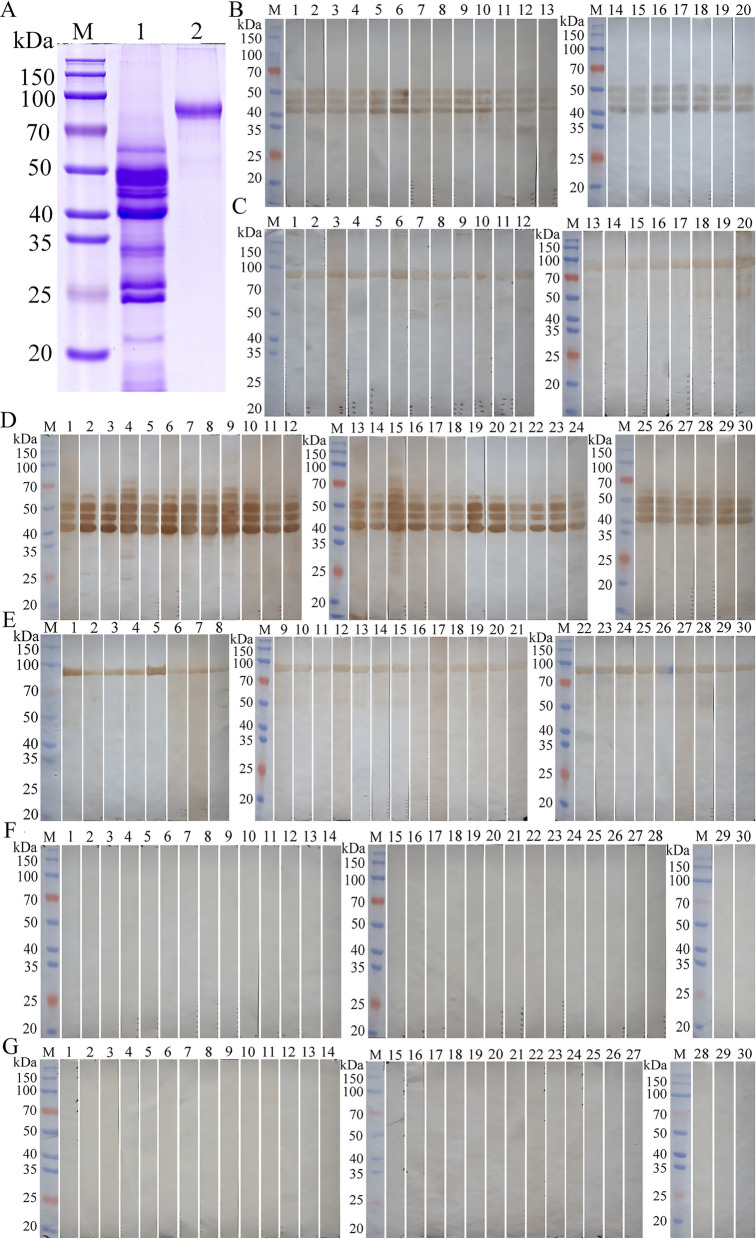
Quality assessment of rTsTryp and ES antigens by western blot. ML ES antigens and rTsTryp were identified by western blot using *T. spiralis*-infected murine serum collected at 14 and 35 dpi, respectively. **a** SDS-PAGE assay of rTsTryp and ML ES antigens. Lanes: M, Protein marker; 1, ML ES antigens; 2, rTsTryp. **b** Western blot of ES antigens using 20 murine infection serum samples collected at 14 dpi (lanes 1–20), showing recognition of three main bands (49.1, 44.5, 40.6 kDa) of ES antigens. **c** Western blot of rTsTryp using 20 murine infection serum samples collected at 14 dpi (lanes 1–20), showing recognition of one distinct band of rTsTryp at 91.6 kDa. **d** Western blot of ES antigens using 30 murine infection serum samples collected at 35 dpi (lanes 1–30), showing recognition of four distinct bands (60, 49.1, 44.5, 40.6 kDa) of ES antigens. **e** Western blot of rTsTryp using 30 murine infection serum samples collected at 35 dpi (lanes 1–30), showing one distinct rTsTryp band at 91.6 kDa that was more evident than shown in** c**.** f**,** g** rTsTryp (**f**) and ML ES antigens (**g**) were not recognized by 30 uninfected murine serum samples. dpi, Days post-infection; ES, excretory/secretory; ML, muscle larvae; rTsTryp, recombinant *T. spiralis* trypsin; SDS-PAGE, sodium dodecyl sulfate-polyacrylamide gel electrophoresis

Prior to performing the ELISA, we assessed the quality of rTsTryp and ML ES antigens by western blot using *T. spiralis*-infected murine sera collected at 14 and 35 dpi. The rTsTryp and ML ES antigens were first separated by SDS-PAGE, and visualization of the products showed a clear individual band of 91.6 kDa for rTsTryp and nine clear bands of 60.0, 49.1, 44.5, 40.6, 33.6, 32.4, 26.9, 24.7 and 21.3 kDa, respectively, for the ML ES antigens (Fig. 7a). These distinct bands of rTsTryp and ES antigens were authenticated by murine infection sera collected at 14 and 35 dpi. Using 20 infection serum samples collected at 14 dpi, three main bands (49.1, 44.5 and 40.6 kDa) of ES antigens were identified, with an identical band pattern in all serum samples (Fig. 7b); also, one distinct band of rTsTryp at 91.6 kDa was identified by the 20 infection serum samples collected at 14 dpi (Fig. 7c). Using 30 murine infection serum samples collected at 35 dpi, the same three main bands (49.1, 44.5, 40.6 kDa) of ES antigens were recognized, but more clearly, as well as an additional band (60 kDa) (Fig. 7D), suggesting that the 60-kDa band may be expressed in the ML stage and that it might be regarded as a diagnostic marker for the muscular phase of *T. spiralis* infection in mice. The distinct band of rTsTryp at 91.6 kDa was also detected by the 30 infection serum samples collected at 35 dpi (Fig. 7e). In contrast, rTsTryp and ML ES antigens were not recognized by 30 uninfected normal murine serum samples (Fig. 7f, g). The results indicated that the rTsTryp and ML ES antigens prepared in this study were of good quality and that the band pattern identified by the infection serum had good reproducibility.

### Detection of* Trichinella*-specific IgG in infected murine serum

*Trichinella-*specific IgG levels in serum samples from mice infected with 200 *T. spiralis* ML and other parasites were measured using the rTsTryp-ELISA and ES antigens-ELISA (Table 1). In serum collected from mice infected with 200 *T. spiralis* ML at 30 dpi, the positive rate of specific IgG was 100% by the two antigens. The two antigens had no cross-reaction with serum collected from mice infected with 30 *A. cantonensis* third-stage larvae, 200 *C. hepatica* infective eggs, 60 *C. sinensis* metacercaria, 100 *S. japonicum* cercariae, and 4 × 10^4 ^*T. gondii* (RH strain) tachyzoites, although the ES antigens had a cross-reaction with one serum sample (3.33%) collected from *S. mansoni* plerocercoid-infected mice. However, the subsequent confirmatory test proved that this one positive serum in the ES-ELISA test was negative on western blot analysis (Additional file 2: Figure S2), indicating that the ES-ELISA had one false positive with murine sparganum infection serum.

**Table 1:** Anti-*Trichinella* immunoglobulin G levels in serum samples collected from mice infected with *T. spiralis* and other parasites, by the recombinant *T. spiralis* trypsin-enyzme-linked immunosorbent assay (ELISA) and the excretory/secretory antigens-ELISA

Worm species^a^	No. of serum samples^b^	rTsTryp-ELISA		ES antigens-ELISA	
		OD value (mean ± SD)	No. of positive samples (%)	OD value (mean ± SD)	No. of positive samples (%)
*Trichinella spiralis*	20	2.01 ± 0.43	20 (100)	2.37 ± 0.22	20 (100)
*Angiostrongylus cantonensis*	12	0.10 ± 0.03	0 (0)	0.07 ± 0.04	0 (0)
*Capillaria hepatica*	10	0.09 ± 0.04	0 (0)	0.08 ± 0.03	0 (0)
*Clonorchis sinensis*	20	0.12 ± 0.04	0 (0)	0.10 ± 0.05	0 (0)
*Schistosoma japonicum*	24	0.14 ± 0.05	0 (0)	0.14 ± 0.05	0 (0)
*Spirometra mansoni*	30	0.09 ± 0.04	0 (0)	0.10 ± 0.05	1 (3.3)
*Toxoplasma gondii*	15	0.04 ± 0.02	0 (0)	0.04 ± 0.02	0 (0)
Uninfected mice	50	0.11 ± 0.02	0 (0)	0.12 ± 0.04	0 (0)

*ES* Excretory/secretory,* OD* optical density,* rTsTryp* recombinant *T. spiralis* trypsin,* SD* standard deviation

^a^The parasite infecting dose per mouse was: 200 *T. spiralis* ML, 30 *A. cantonensis* third-stage larvae, 200 *C. hepatica* infective eggs, 60 *C. sinensis* metacercaria, 100 *S. japonicum* cercariae, 5 *S. mansoni* plerocercoids, and 4 × 10^4^ *T. gondii* (RH strain) tachyzoites

^b^Serum samples of mice infected with *T. spiralis*, *A. cantonensis*, *C. sinensis*, *S. japonicum*, *S. mansoni* and *T. gondii* were collected at 30, 21, 84, 42, 42, 30, and 30 days post-infection, respectively

Seroconversion for positivity of mice experimentally infected with *T. britovi* and *T. nelsoni* was 100% at 35 dpi (Table 2), and seroconversion for positivity of specific IgG in mice infected with *T. nativa* was not significantly different by rTsTryp-ELISA and ES antigens-ELISA (*χ*^2^ = 0.015, *P* = 0.903). However, seropositivity in mice infected with *T. pseudospiralis* was only 42.86% by rTsTryp-ELISA and 34.29% by ES antigens-ELISA (*χ*^2^ = 0.241, *P* = 0.624). Serum samples from *T. pseudospiralis*-infected mice collected at 35 dpi were also tested by the ELISA with *T. pseudospiralis* ML crude antigens. The results showed that the seropositive rate was 100% (35/35) (OD values: 1.99 ± 0.90), while the anti-*Trichinella* IgG level of 50 normal mouse serum samples was negative (OD values: 0.13 ± 0.03), suggesting that the seroconversion had occurred in all *T. pseudospiralis*-infected mice at 35 dpi. These findings suggested that rTsTryp and *T. spiralis* ES antigens were applicable to the serodiagnosis of infection with encapsulated *Trichinella* species, but not to that of infection with non-encapsulated *T. pseudospiralis*.

**Table 2:** Anti-*Trichinella* immunoglobulin G levels in serum samples of mice infected with different *Trichinella* species by the recombinant *T. spiralis* trypsin-enyzme-linked immunosorbent assay (ELISA) and the excretory/secretory antigens-ELISA

*Trichinella* species	No. of serum samples	rTsTryp-ELISA		ES antigens-ELISA	
		OD value (mean ± SD)	No. of positive samples (%)	OD value (mean ± SD)	No. of positive samples (%)
*T. nativa*	23	0.61 ± 0.69	19 (82.6)	0.73 ± 0.70	18 (78.3)
*T. britovi*	14	1.19 ± 0.65	14 (100)	1.63 ± 0.63	14 (100)
*T. nelsoni*	15	1.12 ± 0.56	15 (100)	1.44 ± 0.53	15 (100)
*T. pseudospiralis*	35	0.41 ± 0.51	15 (42.9)	0.32 ± 0.36	12 (34.3)
Uninfected mice	50	0.11 ± 0.02	0	0.12 ± 0.04	0

*ES* Excretory/secretory,* OD* optical density,* rTsTryp* recombinant *T. spiralis* trypsin,* SD* standard deviation

### Dynamic changes of serum specific IgG and IgM during 2–30 dpi

**Fig. 8:**
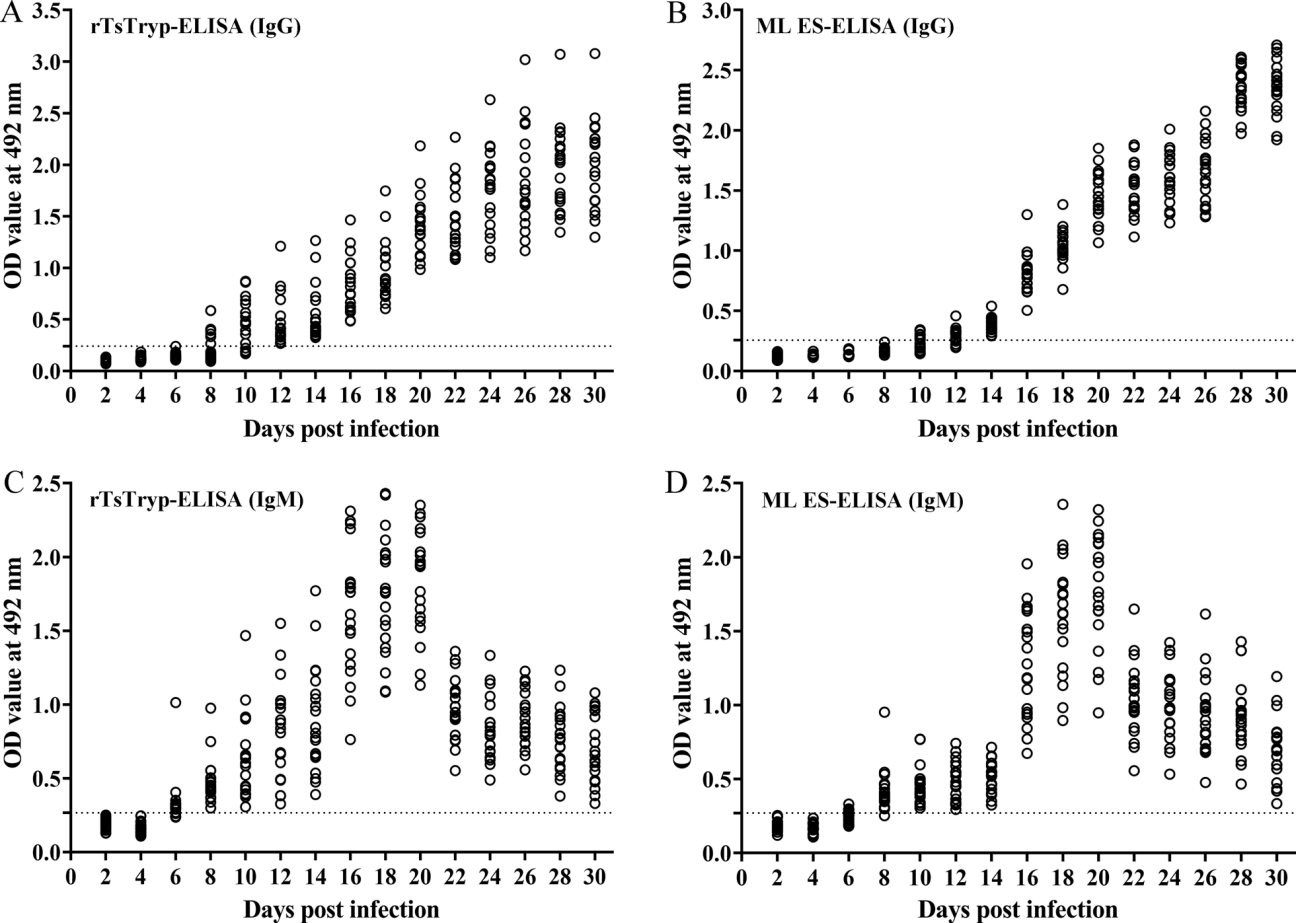
Dynamic changes in serum specific IgG and IgM in serum of mice infected with 20* T. spiralis* larvae collected at 2–30 dpi, detected by ELISA.** a**,** b** Specific IgG levels in serum of infected mice assayed by rTsTryp-ELISA (**a**) and ES antigens-ELISA (**b**).** c**,** d** Serum specific IgM levels of infected mice assayed by rTsTryp-ELISA (**c**) and ES antigens-ELISA (**d**). The dashed lines in the figures are the cutoff values of IgG and IgM. dpi, Days post-infection; ELISA, enzyme-linked immunosorbent assay; ES, excretory/secretory; IgG/IgM, immunoglobulin G/M; OD, optical density; rTsTryp, recombinant *T. spiralis* trypsin

We used 20 mice experimentally infected with 200 *T. spiralis* ML to measure *Trichinella*-specific IgG and IgM levels in serum samples collected at 2–30 dpi by rTsTryp-ELISA and ES antigens-ELISA. Specific IgG was first detected in 35% (7/20) of the infected mice at 8 dpi by rTsTryp-ELISA and in 40% (8/20) of infected mice at 10 dpi by ES antigens-ELISA (*χ*^2^ = 0.107,* P* = 0.744). Anti-*Trichinella* IgG seroconversion reached 100% (20/20) at 12 dpi by rTsTryp-ELISA and 14 dpi by ES-ELISA (Fig. 8a, b), and persisted to 30 dpi. At 6 dpi, serum specific IgM was first detected in 75% (15/20) of infected mice by rTsTryp-ELISA and in 35% (7/20) of infected mice by ES-ELISA (*χ*^2^ = 6.465, *P* = 0.011). Detection of specific IgM reached 100% (20/20) at 8 dpi by rTsTryp-ELISA and at 10 dpi by ES antigens-ELISA (Fig. 8c, d). Anti-*Trichinella* IgM reached peak levels in serum collected at 18–20 dpi, but began to decrease significantly in serum collected on 22 dpi and onwards, although it was still positive by two antigens in serum collected at 30 dpi. These results indicated that the specific antibodies IgG and IgM were detected by rTsTryp 2 days earlier than the ES antigen.

**Fig. 9:**
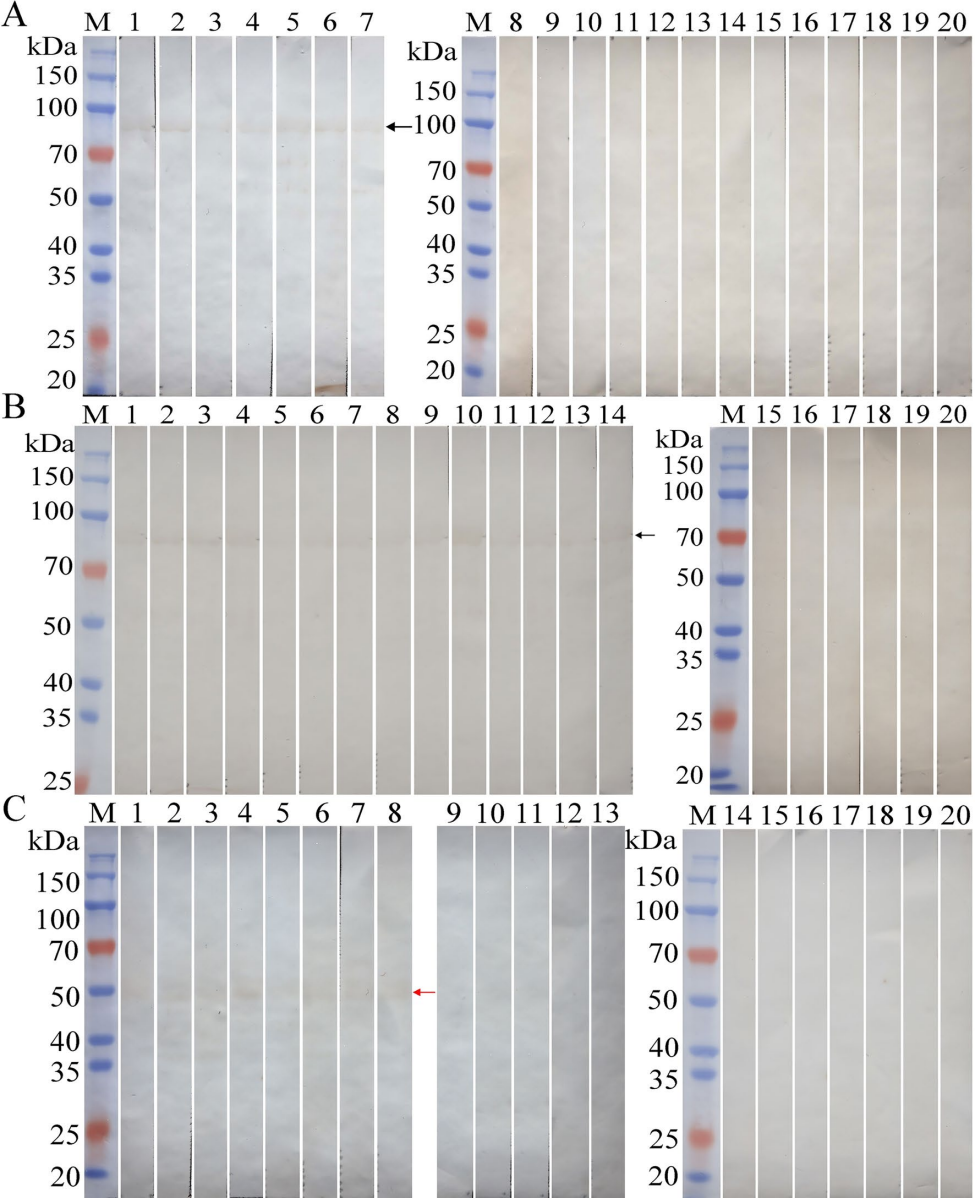
Western blot confirmation of positive serum in the ELISA test. **a** Western blot confirmation of positive serum at 8 dpi according to the rTsTryp-ELISA test. Seven positive serum samples (lanes 1–7) and 13 negative serum samples (lanes 8–20) in the rTsTryp-ELISA test were verified to be positive and negative, respectively, by rTsTryp-Western blot. **b** Western blot confirmation of positive serum at 10 dpi according to the rTsTryp-ELISA test. The 14 positive serum samples (lanes 1–14) and 6 negative serum samples (lanes 15–20) in the rTsTryp-ELISA test proved to be positive and negative, respectively, by rTsTryp-western blot. **c** Western blot confirmation of positive serum at 10 dpi in the ES-ELISA test. The 8 positive serum samples (lanes 1–8) and 12 negative serum samples (lanes 9–20) according to the ES-ELISA test were validated to be weakly positive and negative, respectively, by ES-western blot. All of the positive bands of rTsTryp and ES antigens recognized by western blot with early infection serum were indistinct and weak. The black arrow indicates the rTsTryp band of 91.6 kDa recognized by infection sera; the red arrow indicates the ES antigen band of 49.1 kDa identified by murine infection serum at 10 dpi. dpi, Days post-infection; ELISA, enzyme-linked immunosorbent assay; ES, excretory/secretory; M, protein marker; rTsTryp, recombinant *T. spiralis* trypsin

In order to further verify the accuracy of the ELISA test, we used western blot with two antigens to confirm the anti-*Trichinella* IgG positive serum according to the ELISA test. The results are shown in Fig. 9a. Seven serum samples collected at 8 dpi that were positive in the rTsTryp-ELISA test (negative in the ES-ELISA test) were proved to be positive by western blot with rTsTryp; 13 negative serum samples at 8 dpi were still negative according to western blot with rTsTryp. Western blot with rTsTryp showed that at 10 dpi, 14 positive serum samples in the rTsTryp-ELISA test were verified to be positive, whereas six negative serum samples in the rTsTryp-ELISA test were still negative (Fig. 9b). Positive serum at 10 dpi according to the ES-ELISA test was also evaluated by western blot with ES antigens; the results revealed that eight positive serum samples at 10 dpi in the ES-ELISA test were validated to be weakly positive, while the 12 negative serum samples were still negative (Fig. 9c). These results demonstrated that the detection of *Trichinella*-specific IgG in the serum of infected mice was the same by ELISA and western blot. However, the positive bands of rTsTryp (91.6 kDa) and ES antigens (49.1 kDa) identified by infection sera at 8–10 dpi were indistinct and weak on western blot.

### *Trichinella*-specific IgG in serum of* T. spiralis*-infected pigs

Specific IgG levels in pigs experimentally infected with *T. spiralis* were measured using rTsTryp-ELISA and ES antigens-ELISA. Seropositivity rate in serum of *T. spiralis-*infected swine collected at 70 dpi was 100% by rTsTryp-ELISA and ES-ELISA (Table 3). There was no cross-reaction with serum from pigs infected with *A. suum* and normal porcine sera by two antigens.

**Table 3:** Anti-*Trichinella* immunoglobulin G levels in serum of *T. spiralis*-infected pigs

Parasite species	No. of serum samples^a^	rTsTryp-ELISA		ES antigens-ELISA	
		OD value (mean ± SD)	No. of positive samples (%)	OD value (mean ± SD)	No. of positive samples (%)
*Trichinella spiralis*	18	0.72 ± 0.11	18 (100)	0.93 ± 0.12	18 (100)
*Ascaris suum*	4	0.18 ± 0.03	0	0.22 ± 0.02	0
Uninfected pigs	55	0.13 ± 0.04	0	0.13 ± 0.06	0

*ELISA* Enzyme-linked immunosorbent assay, *ES* excretory/secretory,* OD* optical density,* rTsTryp* recombinant *T. spiralis* trypsin,* SD* standard deviation

^a^*T. spiralis*-infected pig serum samples were obtained at 70 days post-infection. *A. suum* infection serum samples were obtained from naturally infected pigs slaughtered at the slaughterhouse

### Serum anti-*Trichinella* IgG in trichinellosis patients

The sensitivity of the rTsTryp-ELISA and ES antigens-ELISA to detect serum *Trichinella*-specific IgG was 98.1% (51/52 samples) and 94.2% (49/52), respectively (*χ*^2^ = 1.040, *P* = 0.308) in trichinellosis patients. The sensitivity of the two antigens was 100% (52/52) for late trichinellosis patients at 35 dpi. The sensitivity of rTsTryp and ES antigens was 95.00% (19/20 samples) and 85.00% (17/20), respectively (*χ*^2^ = 1.111, *P* = 0.292) for serum samples collected from early trichinellosis patients at 19 dpi (Table 4). The specificity of rTsTryp was 98.7% (235/238), which was significantly higher than the 95.4% (227/238) of ES antigens (*χ*^2^ = 4.710, *P* = 0.030). rTsTryp did not cross-react with serum of patients with other parasitosis (hepatic capillariasis, filariasis, ancylostomiasis, paragonimiasis, schistosomiasis and sparganosis) or with serum from healthy individuals; but it did cross-react with one serum sample from one patient with echinococcosis, clonorchiasis or cysticercosis, respectively (Additional file 3: Figure S3). These findings demonstrated that the sensitivity of rTsTryp and ES antigens was not significant different, but the specificity of rTsTryp was remarkably higher than that of the ES antigens.

**Table 4:** Anti-*Trichinella* immunoglobulin G levels in serum from trichinellosis patients by the recombinant *T. spiralis* trypsin-enyzme-linked immunosorbent assay (ELISA) and the excretory/secretory antigens-ELISA

Sera of patients with	No. of serum samples	rTsTryp-ELISA		ES antigens-ELISA	
		OD value (mean ± SD)	No. of positive samples (%)	OD value (mean ± SD)	No. of positive samples (%)
Trichinellosis	52	0.91 ± 0.37	51 (98.1)	0.97 ± 0.34	49 (94.2)
Early trichinellosis^a^	20	0.95 ± 0.38	19 (95)	0.92 ± 0.48	17 (85)
Late trichinellosis^b^	32	0.89 ± 0.36	32 (100)	1.00 ± 0.21	32 (100)
Hepatic capillariasis	5	0.06 ± 0.02	0	0.08 ± 0.02	0
Filariasis	12	0.24 ± 0.06	0	0.26 ± 0.07	0
Ancylostomiasis	3	0.08 ± 0.05	0	0.11 ± 0.05	0
Paragonimiasis	25	0.15 ± 0.06	0	0.16 ± 0.09	1 (4)
Schistosomiasis	30	0.19 ± 0.07	0	0.26 ± 0.13	2 (6.7)
Clonorchiasis	11	0.21 ± 0.11	1 (9.1)	0.23 ± 0.12	1 (9.1)
Cysticercosis	20	0.21 ± 0.08	1 (5)	0.31 ± 0.16	3 (15)
Echinococcosis	24	0.20 ± 0.11	1 (4.2)	0.28 ± 0.14	2 (8.3)
Sparganosis	8	0.19 ± 0.07	0	0.26 ± 0.19	2 (25)
Healthy individuals	100	0.17 ± 0.08	0	0.20 ± 0.09	0

*ELISA* Enzyme-linked immunosorbent assay, *ES* excretory/secretory,* OD* optical density,* rTsTryp* recombinant *T. spiralis* trypsin,* SD* standard deviation

^a^Early trichinellosis: serum samples of trichinellosis patients were obtained at 19 days post-infection

^b^Later trichinellosis: serum samples of trichinellosis patients were obtained at 35 days post-infection

## Discussion

Trypsin is an important serine protease of the chymotrypsin subfamily that plays a critical role in catalyzing protein hydrolysis [64]. Results from previous studies indicated that serine proteases actively participate in the invasion and pathogenic process of some parasites [65]. Serine proteases have been found to be the most plentiful proteinases in ES proteins at different stages of *T. spiralis* development [39, 66, 67]. These stage-specific serine proteases elicit distinct immune responses during *T. spiralis* infection [68, 69]. In previous studies, we found that the expressions of serine proteases clearly increased in the IIL phase compared to the ML phase [70], suggesting that serine proteases were involved in the IIL intrusion of the gut mucosa. The serine proteases highly expressed in the enteral IIL phase are exposed early to the host’s local intestinal mucosal and systematic immune system, thereby eliciting the production of intestinal IIL stage-specific antibodies; consequently, serine proteases may represent potential early diagnostic antigens of trichinellosis [66].

In the present study, rTsTryp was expressed and purified. As expected, rTsTryp protein exhibited good immunogenicity, and the immunization of mice with rTsTryp elicited an rTsTryp-specific antibody response with high IgG titer. Western blot results revealed that rTsTryp was recognized by both anti-rTsTryp serum and infection serum. While some native TsTryp at 31.0–71.6 kDa in ES antigens of *T. spiralis* ML, IIL and 3-day AW were identified by anti-rTsTryp serum, this is possibly explained by: (i) TsTryp belonging to the superfamily of serine proteases, and diverse serine proteases have the similar functional domains and common epitopes; (ii) TsTryp having two trypsin-like domains [71]; and (iii) TsTryp possessing various isoforms, post-translational modifications and protein processing [8, 31, 72, 73].

The results of qPCR and western blot showed that TsTryp protein was expressed in all *T. spiralis* worm phases (ML, IIL, AW and NBL), although its expression level was distinctly higher in the intestinal stages (IIL, AW). The IFT results demonstrated that TsTryp was distributed on the surface of IIL- and AW-stage worms, and principally localized in the cuticle and stichosome of the nematode; western blot also revealed that several native TsTryp in ES antigens of the nematode was recognized by anti-rTsTryp serum and infection serum. These findings indicated that TsTryp is a surface and secretory protein that is highly expressed in worms of this parasite at the enteral stage. Previous studies showed that other serine proteases of *T. spiralis* (TsSP, TsSP1.1 and TsSP-1.2) expressed in different worm stages also participated in larval invasion [37, 67, 74]. The results of the current study suggested that TsTryp is a serine protease expressed early during infection and that it may play a significant role in invading gut mucosa and inducing the antibody response at the intestinal stage of *T. spiralis* infection.

Serological methods are currently in wide use for the detection of the specific antibody IgG in *Trichinella* infection, with ELISA and western blot with ML ES antigens being the most commonly used serological tests [13]. The ES antigens-ELISA is usually used for the preliminary screening of *Trichinella* infection, whereas western blot is used as a confirmatory test to verify or exclude the non-negative results in the ES antigens-ELISA test [75–77]. In the present study, the quality of rTsTryp was first assessed by western blotting using murine infection sera. The results showed that one clear rTsTryp band (91.6 kDa) and three ES antigen bands (49.1, 44.5 and 40.6 kDa) were distinctly recognized by murine infection serum collected at 14 dpi. An additional band (60 kDa) of ES antigens was also ultimately recognized by murine infection serum collected at 35 dpi, suggesting that this 60-kDa band might be expressed only at the ML stage, and that it is possibly a diagnostic marker for the muscular phase of *T. spiralis* infection. The ES antigen band pattern recognized by murine infection serum was similar to those recognized by swine infection sera [77]. Although the ML ES antigens had higher specificity than the ML somatic crude antigens, different bands (40–70 kDa) are usually recognized by infection serum on western blot [11]. Small variations in the molecular weight of the bands did not affect the identification of infection sera, but careful observation of the protein migration distances is required [78]. rTsTryp always had a distinct and single band at 91.6 kDa on western blot with infection sera collected at different times post infection, demonstrating that the visual observation of the western blot band pattern of rTsTryp was more simple and obvious compared to that of the ML crude and ES antigens [22, 63].

In *T. spiralis*-infected mice, anti-*Trichinella* IgM reached peak levels at 18–20 dpi, followed by a significant decrease beginning at 22 dpi; this trend suggests that *Trichinella*-specific IgM is only useful for the diagnosis of early trichinellosis [15, 79]. The specific IgG seroconversion reached 100% at 12 dpi by the rTsTryp-ELISA and at 14 dpi by the ES-ELISA. Moreover, rTsTryp had good specificity and did not cross-react with sera from uninfected normal mice and mice infected with other parasites (*A. cantonensis*,* Capillaria hepatica*, *Clonorchis sinensis*, *Schistosoma japonicum, Spirometra mansoni* and *T. gondii*)*.* The reason for the low sensitivity of rTsTryp-ELISA and ES-ELISA to detect *T. pseudospiralis* infection sera may be due to the distant phylogeny between encapsulated and non-encapsulated *Trichinella* species. The similarity of the amino acid sequences of trypsin of *T. pseudospiralis* and *T. spiralis* was only 71.4%. The encapsulated and non-encapsulated *Trichinella* species belong to two different clades in the phylogenetic relationship. It may be that TsTryp has more common antigenic epitopes among encapsulated species (*T. nativa*, *T. britovi* and *T. nelsoni*) and fewer common antigenic epitopes in non-encapsulated species (*T. pseudospiralis*). As such, TsTryp might be specific antigen of the encapsulated *Trichinella* species. The findings of this study indicated that the detection time of specific IgG and IgM in infected mice could be advanced by 2 days using rTsTryp; namely, rTsTryp detected specific antibodies 2 days earlier than ES antigens, thereby demonstrating that rTsTryp is superior to the ES antigens for the early diagnosis of *Trichinella* infection. Additionally, only one 49.1-kDa band of ES antigens was recognized by infection murine serum at 10 dpi, which differs from the three bands of ML ES antigens (40–70 kDa) usually recognized by infection serum [80]. It is interesting that on the rTsTryp-western blot, anti-*Trichinella* IgG was also detected 2 days earlier than the ES antigens.

Although the sensitivity of rTsTryp-ELISA for detecting *Trichinella*-specific IgG antibody in serum from patients with early trichinellosis at 19 dpi was higher than that of ES antigens, there was no statistical significance between two antigens, likely due to an insufficient number of samples from these patients. Importantly, the specificity of rTsTryp was significantly higher than of ES antigens. rTsTryp cross-reacted with only one serum sample collected from each of a patient with clonorchiasis, cysticercosis or echinococcosis. The sensitivity and specificity of rTsTryp for detecting *Trichinella*-specific IgG antibody in human serum was similar to that of other recombinant antigens of serine protease family from *T. spiralis* (rTs31, rTsSP and rTsEla) [8, 31, 37] and cystatin-like protein (rCLP) [81]. It was not possible to validate false-positive human serum in the rTsTryp-ELISA test by western blot due to an insufficient number of human serum samples available in our laboratory. Moreover, anti-*Trichinella* IgM levels in serum from trichinellosis patients was not tested due to long-term sample storage logistics. Our results demonstrated that the sensitivity of rTsTryp and ES antigens for detecting *Trichinella*-specific IgG in human serum was not statistically different, but that the specificity of rTsTryp was significantly higher that of the ES antigens. Therefore, rTsTryp could be regarded as an alternative diagnostic antigen for immunodiagnosis of early *T. spiralis* infection. However, the sensitivity, specificity and reproducibility of rTsTryp needs to be further evaluated on a larger sample size of early trichinellosis patients and patients with other helminthic infections and diseases.

The ES antigens-ELISA is the serological test of choice for detecting anti-*Trichinella* IgG in domestic swine due the simplicity of the text as well as low costs and fast detection. Previous studies have shown that ES-ELISA has a higher sensitivity than the digestion of 1 g of muscle sample in pigs with low larval burdens (i.e., < 3 larvae per gram, l pg) [82]. The infection level (inoculation dose) of *T. spiralis* ML in pigs is closely related to the detectable time of serum specific antibodies. In pigs experimentally infected with 25–10,000 larvae, anti-*Trichinella* IgG antibodies were detected at 25–60 dpi, respectively, indicating that the humoral immune response was inoculation dose dependent [83]. However, no direct correlation between antibody level and the ultimate larval burdens in muscle tissues in serologically positive pigs by ELISA has been found [12]. In a previous study, serum *Trichinella*-specific IgG in pigs inoculated with 300 larvae was first detected at 30 dpi by ES antigens-ELISA, which was also verified using western blot [77]. In another study, recombinant *T. spiralis* Serpin was prepared and used to test *T. spiralis*-infected pig serum; the results showed that at 6 weeks after infection, 45 serum samples obtained from 34 pigs infected with various doses (1000, 5000, 10,000 and 30,400 larvae) exhibited 100% positivity in both ES-ELISA and Serpin ELISA [28]. Our results demonstrated that anti-*Trichinella* IgG positivity was 100% at 70 dpi in pigs infected with 5000 larvae using rTsTryp-ELISA and ES-ELISA. It was not possible to assay early swine infection sera by the two antigens in the present study. Therefore, from a veterinary practical point of view, early swine infection serum collected at various time points post-infection should be tested using rTsTryp-ELISA in future studies.

*Trichinella spiralis* contains phenotypically distinct developmental stages throughout its life-cycle (ML, IIL, AW and NBL). The IIL molt 4 times and grow into the AW within 10–30 hpi. Cuticle replacement occurs in each molting process and in larvae emerging from the cuticle of the previous stage [40]. The outer cuticle of each *T. spiralis* stage expresses different protein molecules during larval growth and the molting process, so each worm stage has its stage-specific antigens [25, 84, 85]. Therefore, at the same time as ensuring specificity, the preparation and mixed use of two or three stage-specific recombinant antigens, namely recombinant stage-specific antigens from early intestinal stages (IIL1-4 or AW), will greatly increase the sensitivity of detecting specific antibodies and enable the development of a more accurate early serodiagnosis of *Trichinella* infection [13].

In conclusion, TsTryp was present at various *T. spiralis* developmental stages, with significantly higher levels expressed at the IIL and adult stages, and was primarily distributed in the cuticle and stichosome of this nematode. rTsTryp is an ES antigen with good immunogenicity. Compared with the widely used ML ES antigens, rTsTryp has a similar sensitivity but higher specificity for detecting anti-*Trichinella* IgG. Therefore, rTsTryp is a valuable tool to detect serum specific antibodies against *Trichinella*, and it may be a promising alternative antigen for the serodiagnosis of *Trichinella* infection.

### Supplementary Information

**Additional file 1: Figure S1.** Multiple alignment of peptide sequences of TsTryp with trypsin of other species/genotypes of *Trichinella*. The sequence of TsTryp (XM_003381619.1) was aligned with trypsin of other *Trichinella* species/genotypes*.* The Clustal W analysis in BioEdit software was used to compare peptide sequences of trypsin within the genus *Trichinella.* The black background sequence represents the same part of TsTryp sequence with other species, the gray background sequence represents conservative substitutive residues and numbers behind the sequence represent percentage similarity with the TsTryp sequence.

**Additional file 2: Figure S2.** Western blot confirmation of one false positive serum of sparganum-infected mice in the ES-ELISA test. **A** Analysis of ML ES antigens by SDS-PAGE. Lanes: M, Protein marker; 1, muscle larvae ES antigens. **B** Western blot confirmation of one false ES-ELISA positive serum. Lanes: M, Protein marker; 1, muscle larvae ES antigens recognized using *T. spiralis*-infected murine serum; 2, muscle larvae ES antigens not recognized using uninfected mouse serum; 3, muscle larvae ES antigens were not identified using sparganum-infected mouse serum which was false positive in ES-ELISA test.

**Additional file 3: Figure S3.** Scatter plot of absorbance values at 492 nm of rTsTryp-ELISA (**A**) and ES antigens-ELISA (**B**) for testing specific IgG in sera from patients infected with *Trichinella* and other parasites. Horizontal dashed lines are cutoff values from rTsTryp-ELISA and ES antigens ELISA.

## Data Availability

The data supporting the conclusion of this research have been included within the article.

## Abbreviations

AW: Adult worms
dpi: Days post-infection
ECL: Enhanced chemiluminescence
ELISA: Enzyme-linked immunosorbent assay
ES: Excretory/secretory
hpi: Hours post-infection
ICT: International Commission on Trichinellosis
IFT: Immunofluorescence test
IIL: Intestinal infectious larvae
ML: Muscle larvae
NBL: Newborn larvae
NJ: Neighbor-joining
PBST: Phosphate-buffered saline containing 0.05% Tween 20
pI: Isoelectric point
(r)TsTryp: (Recombinant) *T. spiralis* trypsin
TBST: Tris-buffer saline containing 0.05% Tween 20
