# Supplementary figures and images for: Application of a recombinant novel trypsin from Trichinella spiralis for serodiagnosis of trichinellosis
Source: Parasit Vectors. 2024 Jan 4;17:9. doi: 10.1186/s13071-023-06067-7 (PMC10768479; doi:10.1186/s13071-023-06067-7)

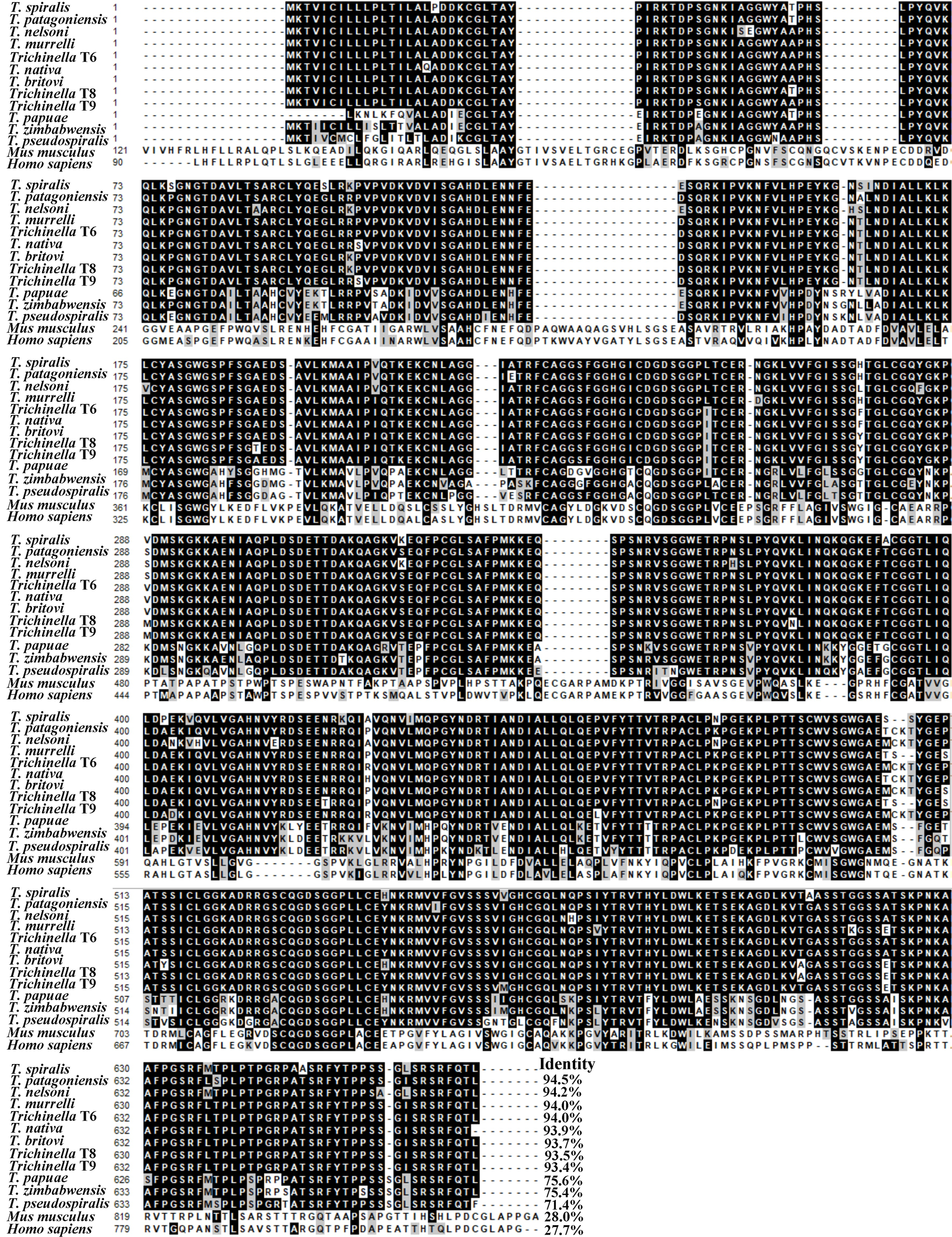

Supplement: Supplementary file 1 — Additional file 1: Figure S1. Multiple alignment of peptide sequences of TsTryp with trypsin of other species/genotypes of Trichinella. The sequence of TsTryp (XM_003381619.1) was aligned with trypsin of other Trichinella species/genotypes. The Clustal W analysis in BioEdit software was used to compare peptide sequences of trypsin within the genus Trichinella. The black background sequence represents the same part of TsTryp sequence with other species, the gray background sequence represents conservative substitutive residues and numbers behind the sequence represent percentage similarity with the TsTryp sequence. [file 13071_2023_6067_MOESM1_ESM.tif]

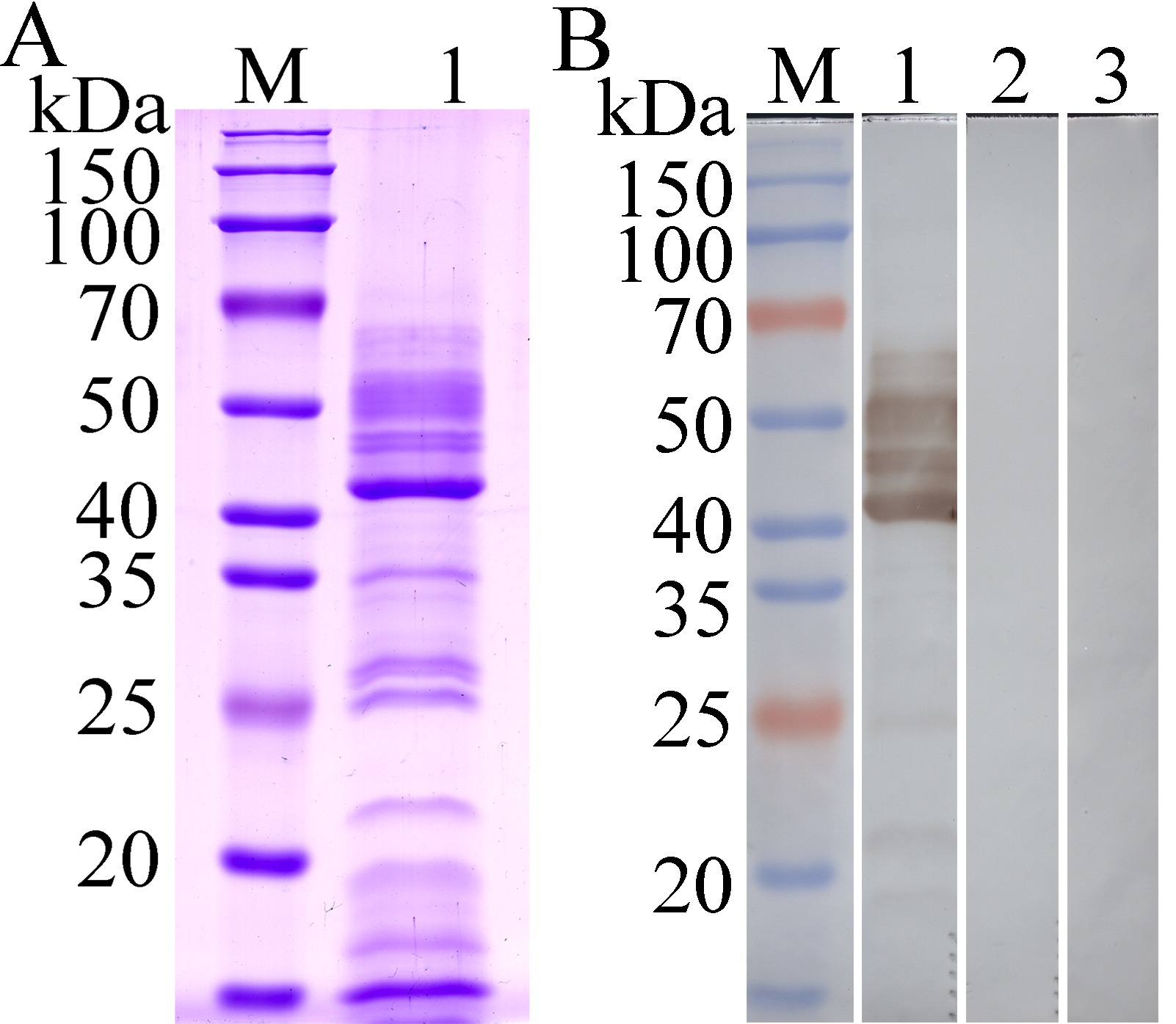

Supplement: Supplementary file 2 — Additional file 2: Figure S2. Western blot confirmation of one false positive serum of sparganum-infected mice in the ES-ELISA test. A Analysis of ML ES antigens by SDS-PAGE. Lanes: M, Protein marker; 1, muscle larvae ES antigens. B Western blot confirmation of one false ES-ELISA positive serum. Lanes: M, Protein marker; 1, muscle larvae ES antigens recognized using T. spiralis-infected murine serum; 2, muscle larvae ES antigens not recognized using uninfected mouse serum; 3, muscle larvae ES antigens were not identified using sparganum-infected mouse serum which was false positive in ES-ELISA test. [file 13071_2023_6067_MOESM2_ESM.tif]

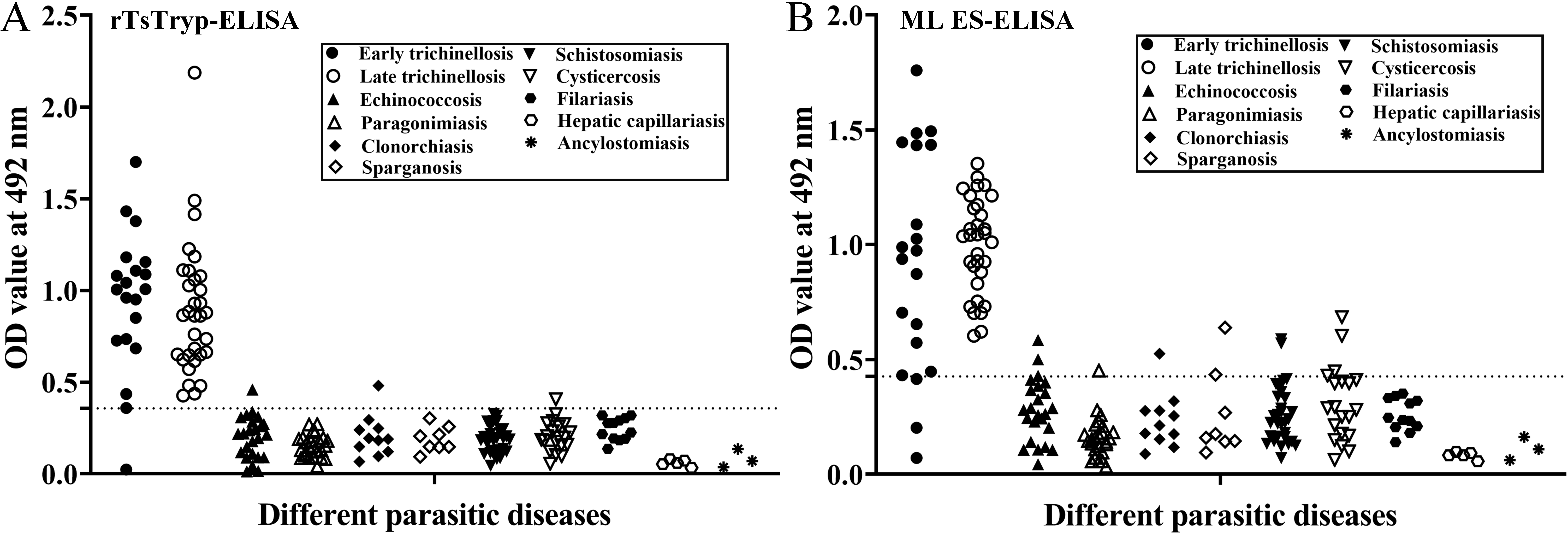

Supplement: Supplementary file 3 — Additional file 3: Figure S3. Scatter plot of absorbance values at 492 nm of rTsTryp-ELISA (A) and ES antigens-ELISA (B) for testing specific IgG in sera from patients infected with Trichinella and other parasites. Horizontal dashed lines are cutoff values from rTsTryp-ELISA and ES antigens ELISA. [file 13071_2023_6067_MOESM3_ESM.tif]
